# Quality of life of the Indonesian general population: Test-retest reliability and population norms of the EQ-5D-5L and WHOQOL-BREF

**DOI:** 10.1371/journal.pone.0197098

**Published:** 2018-05-11

**Authors:** Fredrick Dermawan Purba, Joke A. M. Hunfeld, Aulia Iskandarsyah, Titi Sahidah Fitriana, Sawitri S. Sadarjoen, Jan Passchier, Jan J. V. Busschbach

**Affiliations:** 1 Department of Psychiatry, Section Medical Psychology and Psychotherapy, Erasmus MC University Medical Center, Rotterdam, The Netherlands; 2 Department of Developmental Psychology, Faculty of Psychology, Universitas Padjadjaran, Jatinangor, Indonesia; 3 Department of Clinical Psychology, Faculty of Psychology, Universitas Padjadjaran, Jatinangor, Indonesia; 4 Faculty of Psychology, YARSI University, Jakarta, Indonesia; 5 Department of Clinical, Neuro & Developmental Psychology, VU University, Amsterdam, The Netherlands; National University of Singapore, SINGAPORE

## Abstract

**Objectives:**

The objective of this study is to obtain population norms and to assess test-retest reliability of EQ-5D-5L and WHOQOL-BREF for the Indonesian population.

**Methods:**

A representative sample of 1056 people aged 17–75 years was recruited from the Indonesian general population. We used a multistage stratified quota sampling method with respect to residence, gender, age, education level, religion and ethnicity. Respondents completed EQ-5D-5L and WHOQOL-BREF with help from an interviewer. Norms data for both instruments were reported. For the test-retest evaluations, a sub-sample of 206 respondents completed both instruments twice.

**Results:**

The total sample and test-retest sub-sample were representative of the Indonesian general population. The EQ-5D-5L shows almost perfect agreement between the two tests (Gwet’s AC: 0.85–0.99 and percentage agreement: 90–99%) regarding the five dimensions. However, the agreement of EQ-VAS and index scores can be considered as poor (ICC: 0.45 and 0.37 respectively). For the WHOQOL-BREF, ICCs of the four domains were between 0.70 and 0.79, which indicates moderate to good agreement. For EQ-5D-5L, it was shown that female and older respondents had lower EQ-index scores, whilst rural, younger and higher-educated respondents had higher EQ-VAS scores. For WHOQOL-BREF: male, younger, higher-educated, high-income respondents had the highest scores in most of the domains, overall quality of life, and health satisfaction.

**Conclusions:**

This study provides representative estimates of self-reported health status and quality of life for the general Indonesian population as assessed by the EQ-5D-5L and WHOQOL-BREF instruments. The descriptive system of the EQ-5D-5L and the WHOQOL-BREF have high test-retest reliability while the EQ-VAS and the index score of EQ-5D-5L show poor agreement between the two tests. Our results can be useful to researchers and clinicians who can compare their findings with respect to these concepts with those of the Indonesian general population.

## Introduction

Health-related quality of life (HRQOL) questionnaires are commonly utilized (i) to monitor perceived health status in epidemiological surveys, (ii) to assess the subjective health and well-being of populations and patients, (iii) to measure outcomes in effectiveness studies, and (iv) in health technology assessment [1]. HRQOL questionnaires can be classified as generic and disease-specific. The former are used to measure HRQOL across all kinds of respondents. The latter are designed to narrow the scope of assessment to the health-related problems in specific diagnosis, treatment, or age groups [2].

There are several generic measures of HRQOL that are widely used in the world, including EQ-5D and WHOQOL-BREF (World Health Organization Quality of Life Scale–Abbreviated form). The EQ-5D-5L instrument, provided by the EuroQol Group, consists of five items covering five health state dimensions: mobility, self-care, usual activities, pain/discomfort, and anxiety/depression [3]. The descriptive system constructed from these dimensions can be converted into an index score by applying health preference weights elicited from a general population. This index score can also be used in economic evaluations to assess the cost-effectiveness of health interventions, and is as such one of the most widely used HRQOL questionnaires in the world [4].

The WHOQOL-BREF instrument, developed by the World Health Organization (WHO), measures four domains of quality of life: physical, psychological, social and environmental with its 26 items. It was devised from a cross-cultural methodology to be used in epidemiological studies and in transcultural investigations [5, 6]. The WHOQOL-BREF presents a differentiated picture of quality of life, addressing social, psychological, physical, and environmental functioning [7].

These two instruments have been proved valid in many contexts, and across many health conditions in many countries [6, 8–16], including Indonesia [17, 18]. In Indonesia, both questionnaires are increasingly being used in different types of investigations, for example in the measurement of quality of life in different patient groups [19–22] and in cost-effectiveness studies [23–25]. Thus far, no investigation has measured the stability over time of both questionnaires when measuring the HRQOL of the Indonesian general population: the test-retest reliability. It would be difficult to defend the use of a quality of life instrument if the results change over time due to its unreliability. Moreover, increasing use of both questionnaires in Indonesia demands the need for normative scores to be used as reference values for various patient groups or any particular group of individuals comparison. This need is particularly felt as in the coming years a new national health insurance system is implemented in the whole of Indonesia, requiring a monitoring system for evaluation of its effect. These general population norms, provide a useful guide to interpret the results of different studies of quality of life. Such population norms are not available in Indonesia. Therefore, the aims of this study were to measure the test-retest reliability of EQ-5D-5L and WHOQOL-BREF and to derive Indonesian adult general population norms for both instruments according to different socio-demographic characteristics, i.e. residence, gender, age, education level, income, religion, and ethnicity.

## Methods

This study was part of a larger study focused upon the adult general population, in which several questionnaires were tested in a face-to-face setting at the home/office of the interviewers or at the homes of the subjects. The present manuscript is focused on presenting the frequency distribution of the responses on the descriptive part of EQ-5D-5L and WHOQOL-BREF (see below) as obtained in the Indonesian general population. This study must be distinguished from the study in which we ‘valued’ the health states of the EQ-5D with Time Trade-Off (TTO) and Discrete Choice Experiments (DCE) [26] using the same population. The outcome of that study is of interest for the use of the EQ-5D-5L in health economics and Health Technology Assessment. The present study reports on the more classical way of presenting norm score, that is the frequency of the score in the general population. The study was approved by the Health Research Ethics Committee, Faculty of Medicine, Padjadjaran University, Indonesia.

### Sampling and data collection

The details of sampling and interviewers could be found elsewhere [26]. In short, a multistage stratified quota method was utilized with respect to residence (urban/rural), gender (male/female), age (17-30/31-50/above 50), level of education (basic: primary school and below/middle: high school/high: all others), religion (Islam/Christian/Others) and ethnicity (self-declared: Jawa/Sunda/Sumatera/Sulawesi/Madura-Bali/Others). The pre-defined quotas were based on data from the Indonesian Bureau of Statistics [27]. Each respondent received a mug or a t-shirt specifically designed for this study as a token of appreciation.

Sixteen interviewers were hired to collect the data. Data collection was conducted in six cities and their surroundings located in different parts of Indonesia: Jakarta, Bandung, Jogjakarta, Surabaya, Medan, and Makassar. Signed informed consent was obtained from all the respondents.

After the first interview the interviewer asked for a respondent’s consent to be interviewed again (retest). The interval between the first test and the retest ranged from 10 days to a month. The retest interview was held by the same interviewer. The characteristics of the test-retest sub-sample were matched with the Indonesian general population for three factors: residence, gender, and age. The other three characteristics: level of education, religion, and ethnicity, were not matched due to logistical constraints in finding respondents who were suitable and willing to participate in the second interview.

### Instruments

EQ-5D-5L was developed by the EuroQol Group. It assesses HRQOL on five dimensions: mobility (MO), self-care (SC), usual activities (UA), pain/discomfort (PD), and anxiety/depression (AD). Responses are recorded on a 5-point scale indicating levels of severity: no problems, slight problems, moderate problems, severe problems, and unable/extreme problems. This ‘descriptive system’ is followed by a self-rating of overall health status on a visual analogue scale (EQ-VAS) ranging from 0 (“The worst health you can imagine") to 100 (“The best health you can imagine"). Since Bahasa Indonesia is the national and official language that is spoken throughout the country, we used the official EQ-5D-5L Bahasa Indonesia version 1.0 provided by the EuroQol Group. This translation of EQ-5D was produced using a standardized translation protocol [28] and has been proven as a valid and reliable questionnaire to be used in Indonesia [17]. Completion of EQ-5D-5L was undertaken using an online version of the questionnaire, as part of the EuroQol EQ-Valuation Technology (EQ-VT) platform version 2.0.

The WHOQOL-BREF was developed by the WHOQOL Group as a short version of the WHOQOL-100. This instrument comprises 26 questions, two of which measure overall quality of life and general health. The other 24 questions are divided into four domains: physical, psychological, social relationships, and environmental. Each item is scored on a scale from 1 to 5. The scores are then transformed into a linear scale between 0 and 100, with 0 being the least favourable quality of life and 100 being the most favourable [5]. The Indonesian version of the WHOQOL-BREF is available and has been proven as a valid and reliable questionnaire to be used in Indonesia [18]. In line with the manual of the English version of the WHOQOL-BREF [29], we chose to apply a time-frame for the WHOQOL-BREF of four weeks, and our version was acknowledged by the WHO as the revised official Bahasa Indonesia version. We used the self-administered paper-based WHOQOL-BREF for this study.

Demographic data was collected using a questionnaire, which included: name, place and date of birth, ethnicity, religion, education level, work status, monthly income, and marital status.

### Statistical analysis

Categorical data was analyzed using cross-tabulation. Means and standard deviations (SD) were calculated for continuous data. We calculated the test-retest reliability of both questionnaires using the Gwet’s agreement coefficient (Gwet’s AC) test [30]. This test was chosen to tackle the ‘Kappa paradoxes’: i.e. high percentage agreement but low kappa which usually occurs in the sample with low prevalence of cases or problems, such as in general population. Details can be found in the work of Gwet [30] and Wongpakaran [31]. This Gwet’s AC was also used to calculate the test-retest reliability of overall quality of life and general health from WHOQOL-BREF. Percentage of agreement among test and retest were also calculated. Test–retest reliability of the EQ-VAS, the EQ-5D-5L index scores, and the four domains scores of WHOQOL-BREF were evaluated by the intra-class correlation coefficient (ICC, two-way random effects, absolute agreement). When the data is non-normally distributed, we transformed the data: i.e. log, square and cubic transformation, and reapplied the ICC. We applied the following reliability guideline for strength of the ICC values: <0.5 = poor, 0.5–0.75 = moderate, 0.75–0.9 = good, and >0.90 = excellent [32]. Analysis of concordance by Lin’s concordance correlation coefficient (CCC) was conducted to provide additional analysis of non-normally distributed data. In addition, we used the Bland-Altman plots for the EQ-VAS, index scores, and the four domains of WHOQOL-BREF to examine visually the agreement between test and retest scores. To obtain EQ-5D-5L ‘utility’ index scores, the new Indonesian value set was used [26]. For the self-reported health profile obtained from EQ-5D-5L, we calculated the percentage of respondents who responded to each level of each dimension and calculated those percentages across different socio-demographic characteristics, i.e.: residence, gender, age, education level, religion, ethnicity and income. We compared the proportions of self-reported health for the different socio-demographic characteristics with the Chi-square test. For the population norms, the EQ-5D-5L mean scores (i.e. EQ-VAS scores and index scores) and WHOQOL-BREF mean scores (domain scores, overall quality of life, and general health) were calculated across different socio-demographic characteristics. For comparison of scores between two groups (residence and gender), Welch's unequal variances t-test was used, given the skewed data and different variances. ANOVA was used to compare more than two groups: age, education level, religion, ethnicity and income.

All statistical analyses were carried out using the STATA version 13 software.

## Results

### Characteristics of the respondents

In total 1056 of 1117 respondents who were approached completed the two questionnaires. As can be seen in Table 1, the differences between the study sample and the target distribution as provided by the Indonesian Bureau of Statistics were small (< 4%).

**Table 1:** General socio-demographics of the study respondents^a^.

Characteristics	Study Sample N = 1056 (%)		Indonesian population (%)	Differences (%)
Residence				
Rural	507	48.01	46.70	+1.31
Urban	549	51.99	53.30	-1.31
Gender				
Female	528	50.00	49.65	+0.35
Male	528	50.00	50.35	-0.35
Age				
17–30	419	39.68	36.73	+2.95*
31–50	438	41.48	40.76	+0.72
>50	199	18.84	22.51	-3.67*
Education				
Low	340	32.20	35.18	-2.98*
Middle	551	52.18	51.72	+0.46
High	165	15.63	13.10	+2.53*
Religion				
Islam	922	87.31	87.18	+0.13
Christian	103	9.75	9.86	-0.11
Others	31	2.94	2.96	-0.02
Ethnicity				
Jawa	442	41.86	40.22	+1.64
Sunda	200	18.94	15.50	+3.44*
Sumatera	128	12.12	15.02	-2.90*
Sulawesi	63	5.97	8.09	-2.12*
Madura—Bali	52	4.92	4.70	+0.22
Others	171	16.19	16.47	-0.28
Monthly income**				
0–500 (0–35)	515	48.77	-	-
500–2.500 (35–176)	361	34.19	-	-
2.500–5.000 (176–353)	130	12.31	-	-
> 5.000 (>353)	50	4.73	-	-
General characteristics of test-retest respondents				
Characteristics	Test-retest Sample N = 206 (%)		Indonesian population (%)	Differences (%)
Residence				
Rural	103	50.00	46.70	+3.30
Urban	103	50.00	53.30	-3.30
Gender				
Female	103	50.00	49.65	+0.35
Male	103	50.00	50.35	-0.35
Age				
17–30	79	38.35	36.73	+1.62
31–50	86	41.75	40.76	+0.99
>50	41	19.90	22.51	-2.61

*: p-value<0.05

**: Monthly income in thousands of Rupiah and Euro in brackets

^a^This data is also presented, with some slight differences, in Purba et al [26]

### Test-retest reliability

From 227 participants who completed the two questionnaires twice, 21 participants were excluded because the time interval between both interviews (i.e. test-retest) was more than a month, which was considered as too long for a retest interview. Thus, the sample tested numbered 206 respondents. The mean interval between the first and second interviews was 17.45 days (SD = 4.71). The characteristics of the remaining test-retest respondents were similar to those of the Indonesian general population and the total sample in terms of residence, gender and age (see Table 1).

**Fig 1:**
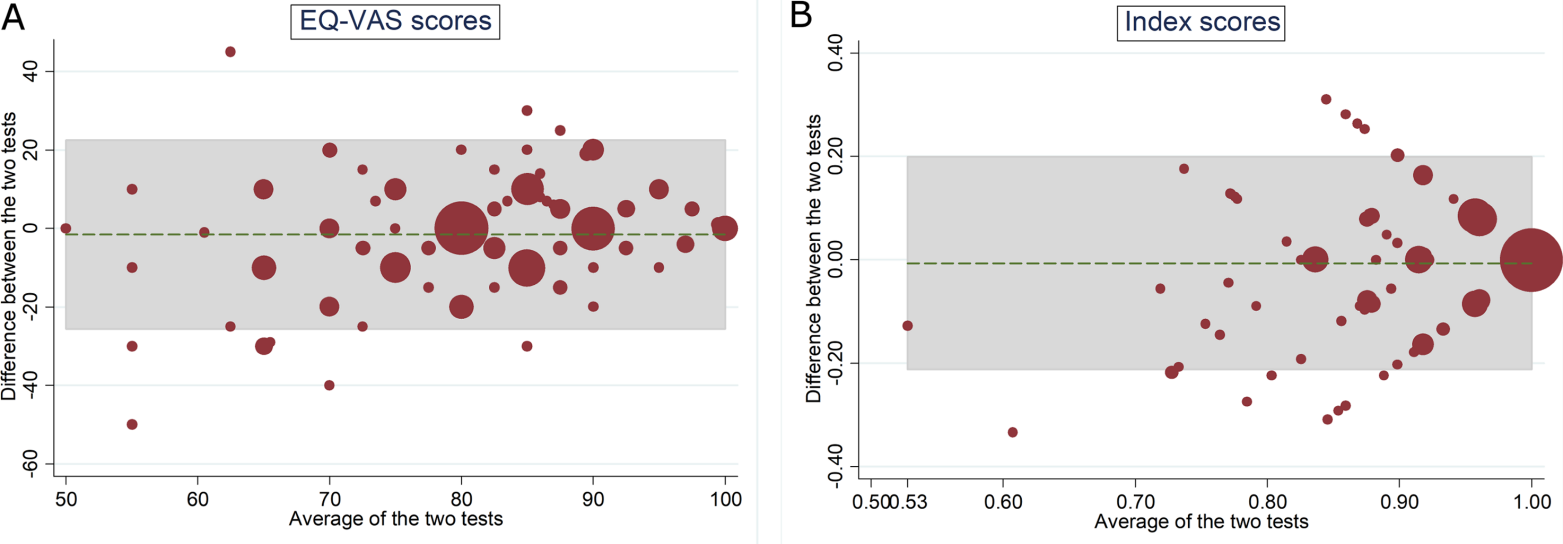
Test-retest Bland-Altman plot of the EQ-5D-5L. (A) VAS scores: 5.3% outside the limit of agreements (B) Index scores: 7.3% Agreement coefficient (AC) of two overall items of WHOQOL-BREF: quality of life and general health, were 0.91 and 0.86, and the percentage agreement were 94.4% and 92.6%, respectively. These indicates almost perfect agreement between test and retest. ICCs of WHOQOL-BREF’ four domains were between 0.70 and 0.79, which indicates moderate to good agreement (see Table 2). The Bland-Altman plot shows that the percentage of data points that lies outside the limits of agreement were 4.9% for the physical and environmental domains, 5.9% for the psychological domain, and 6.3% for the social domain. The majority of these poor agreements data points lies between mean score of 60 to 80. On the other hand, the data points in the lower part (below 60) and higher part (above 80) of the scales were all still located within the limits of agreement (see Fig 2).

The EQ-5D-5L shows almost perfect agreement between the two tests (Gwet’s AC: 0.85–0.99 and percentage agreement: 90–99%) regarding the five dimensions. However, the agreement of EQ-VAS and index scores can be considered to be poor with ICC scores of 0.45 and 0.37 respectively. Transforming the data resulted in small increases only to the ICCs. Similar scores were shown by the concordance correlation analysis. These results can be seen in Table 2. Inspection of the Bland–Altman plot of the EQ-VAS shows that there were 5.3% of data points where agreement is considered as poor: i.e. lies outside the ±1.96 SD limits of agreement. The majority of these data points were from the lower part of the scale: mean score of 70 and less. For the index score, majority of the 7.3% of the poor agreements data points were between the 0.8 and 0.9 mean index score. For the two measures: EQ-VAS and index score, higher agreement between the two tests were shown by respondents with better health: i.e. all the data points of EQ-VAS mean score of 85 and above and between mean index scores of 0.9 and 1.0 were within the limits of agreement (see Fig 1).

**Fig 2:**
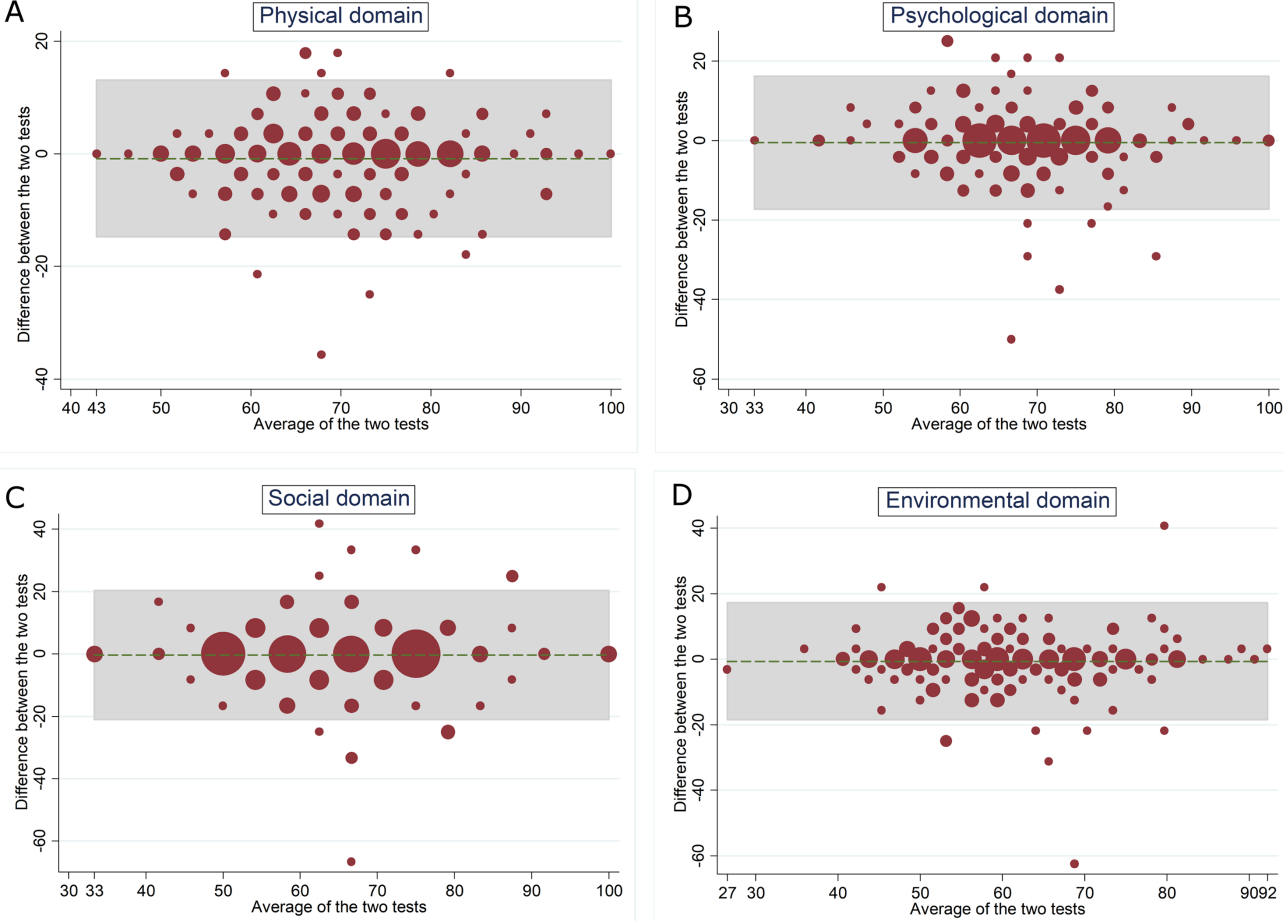
Test-retest Bland-Altman plot of the WHOQOL-BREF. (A) physical domain: 4.9% outside the limit of agreements (B) psychological domain: 5.9% (C) social domain: 6.3% (D) environmental domain: 4.9%.

**Table 2:** Agreement coefficient, percentage agreement, Intraclass correlation coefficient and concordance correlation coefficient.

**EQ-5D-5L**						
Dimension	Agreement coefficient^a^	Percentage Agreement		ICC Non^b^	ICC Transf^c^	CCC^d^
Mobility	0.97	97.45	EQ-VAS	0.45	0.40; 0.48; 0.49	0.45
Self-care	0.99	99.15	Index score	0.37	0.38; 0.36; 0.35	0.37
Usual activities	0.96	96.72				
Pain/Discomfort	0.86	91.02				
Anxiety/depression	0.85	89.93				
**WHOQOL-BREF**						
Domain	Agreement coefficient^a^	Agreement (%)	Domain	ICC	CCC	
Overall QoL	0.91	94.39	Physical	0.79	0.79	
General health	0.86	92.56	Psychological	0.70	0.70	
			Social	0.70	0.70	
			Environmental	0.72	0.72	

^a^ Gwet’s Agreement Coefficient

^b^ Data is not transformed

^c^ Data is transformed: log, squared, and cubic and the results presented following the order of transformation

^d^ Lin’s concordance correlation coefficients

### EQ-5D-5L population norms

EQ-5D-5L population norms were derived from the following: (i) self-reported health profiles, (ii) EQ-VAS scores, and (iii) index scores based on the Indonesian value set.

**Fig 3:**
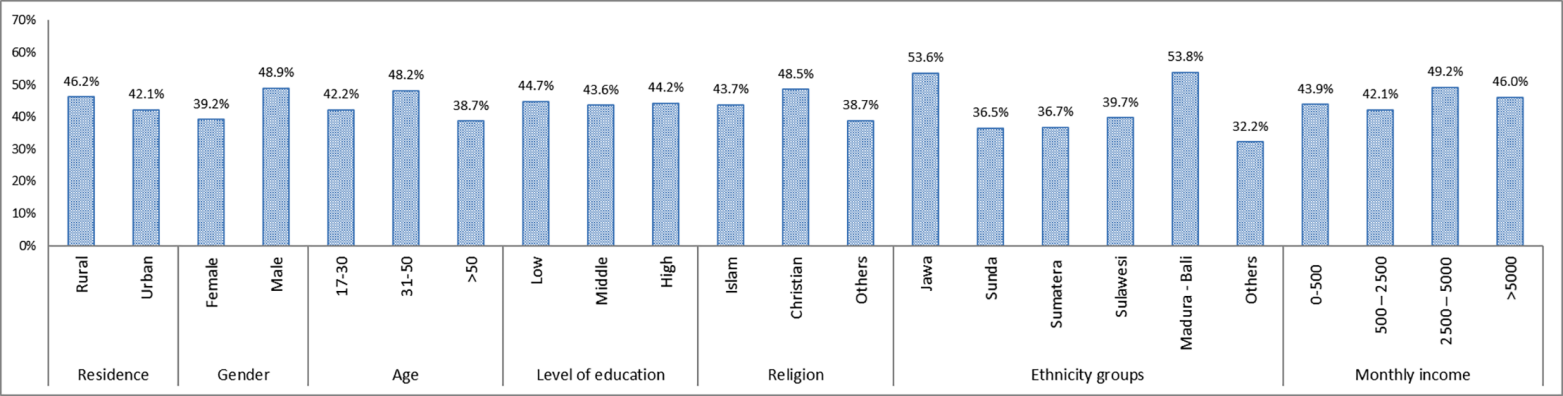
Percentage of respondents reporting no problems on any of the 5 dimensions (health state ‘11111’) (N = 465).

The EQ-5D-5L self-reported health profiles in the total sample and sub-samples by residence, gender, age, education level, religion, ethnicity and monthly income can be seen in Tables 3–7. Nearly half of the samples (44.07%) responded with response pattern ‘11111’: no problems on any of the five dimensions. The proportions of respondents with health state among different demographic characteristics can be seen in Fig 3. The two dimensions with the highest proportions of respondents who reported having problems (level 2–5) were pain/discomfort (39.7%) and anxiety/depression (34.3%), whereas the lowest was in the self-care dimension (1.9%). The proportions of self-reported problems differed between all socio-demographic subsamples for at least one dimension. For instance, females reported having significantly more problems than males in mobility, usual activities and pain/discomfort. Older respondents reported having more problems in all dimensions mobility, self-care, usual activities and pain/discomfort compared to younger ones, while the opposite is shown for the anxiety/depression dimension with more anxiety/depression problems experienced by younger respondents.

**Table 3:** EQ-5D-5L self-reported health profiles in mobility dimension in the total population sample and sub-samples by residence, gender, age, education level, religion, ethnicity and monthly income (%).

	MOBILITY				
Socio-demographic	No problems	Slight Problems	Moderate problems	Severe Problems	Unable/ extreme problems
All respondents	92.05	6.72	1.04	0.19	0.00
Residence					
Rural	93.69*	4.73	1.58	0.00	0.00
Urban	90.53*	8.56	0.55	0.36	0.00
Gender					
Female	89.02*	9.28	1.52	0.19	0.00
Male	95.08*	4.17	0.57	0.19	0.00
Age					
17–30	96.42*	2.86	0.48	0.24	0.00
31–50	92.24*	7.31	0.23	0.23	0.00
>50	82.41*	13.57	4.02	0.00	0.00
Education					
Low	90.59	8.53	0.88	0.00	0.00
Middle	92.20	6.17	1.45	0.18	0.00
High	94.55	4.85	0.00	0.61	0.00
Religion					
Islam	92.08	6.62	1.09	0.22	0.00
Christian	92.23	6.80	0.97	0.00	0.00
Others	90.32	9.68	0.00	0.00	0.00
Ethnicity					
Jawa	95.70*	3.62	0.68	0.00	0.00
Sunda	94.00*	5.00	1.00	0.00	0.00
Sumatera	87.50*	10.94	1.56	0.00	0.00
Sulawesi	84.13*	11.11	4.76	0.00	0.00
Madura—Bali	92.31*	7.69	0.00	0.00	0.00
Others	86.55*	11.70	0.58	1.17	0.00
Monthly income (in thousand Rupiahs)					
0–500	90.87	7.18	1.55	0.39	0.00
500–2500	93.91	5.26	0.83	0.00	0.00
2500–5000	92.31	7.69	0.00	0.00	0.00
>5000	90.00	10.00	0.00	0.00	0.00

* = the proportions of self-reported health in the corresponding dimension between the demographic groups differs statistically significant, p-value <0.05

**Table 4:** EQ-5D-5L self-reported health profiles in self-care dimension in the total population sample and sub-samples by residence, gender, age, education level, religion, ethnicity and monthly income (%).

	SELF-CARE				
Socio-demographic	No problems	Slight Problems	Moderate problems	Severe Problems	Unable/ extreme problems
All respondents	98.11	1.71	0.09	0.09	0.00
Residence					
Rural	97.83	1.78	0.20	0.20	0.00
Urban	98.36	1.64	0.00	0.00	0.00
Gender					
Female	98.11	1.52	0.19	0.19	0.00
Male	98.11	1.89	0.00	0.00	0.00
Age					
17–30	98.57*	1.43	0.00	0.00	0.00
31–50	98.86*	1.14	0.00	0.00	0.00
>50	95.48*	3.52	0.50	0.50	0.00
Education					
Low	97.06	2.65	0.29	0.00	0.00
Middle	98.37	1.45	0.00	0.18	0.00
High	99.39	0.61	0.00	0.00	0.00
Religion					
Islam	98.48*	1.30	0.11	0.11	0.00
Christian	97.09*	2.91	0.00	0.00	0.00
Others	90.32*	9.68	0.00	0.00	0.00
Ethnicity					
Jawa	98.42*	1.58	0.00	0.00	0.00
Sunda	99.00*	1.00	0.00	0.00	0.00
Sumatera	97.66*	1.56	0.78	0.00	0.00
Sulawesi	96.83*	1.59	0.00	1.59	0.00
Madura—Bali	98.08*	1.92	0.00	0.00	0.00
Others	97.08*	2.92	0.00	0.00	0.00
Monthly income (in thousand Rupiahs)					
0–500	98.25*	1.36	0.19	0.19	0.00
500–2500	97.78*	2.22	0.00	0.00	0.00
2500–5000	99.23*	0.77	0.00	0.00	0.00
>5000	96.00*	4.00	0.00	0.00	0.00

* = the proportions of self-reported health in the corresponding dimension between the demographic groups differs statistically significant, p-value <0.05

**Table 5:** EQ-5D-5L self-reported health profiles in usual activities dimension in the total population sample and sub-samples by residence, gender, age, education level, religion, ethnicity and monthly income (%).

	USUAL ACTIVITIES				
Socio-demographic	No problems	Slight Problems	Moderate problems	Severe Problems	Unable/ extreme problems
All respondents	89.20	9.66	1.14	0.00	0.00
Residence					
Rural	89.94	8.68	1.38	0.00	0.00
Urban	88.52	10.56	0.91	0.00	0.00
Gender					
Female	86.74*	11.55	1.71	0.00	0.00
Male	91.67*	7.77	0.57	0.00	0.00
Age					
17–30	88.54*	10.02	1.43	0.00	0.00
31–50	91.78*	7.99	0.23	0.00	0.00
>50	84.92*	12.56	2.51	0.00	0.00
Education					
Low	90.88*	7.94	1.18	0.00	0.00
Middle	87.30*	11.98	0.73	0.00	0.00
High	92.12*	5.46	2.42	0.00	0.00
Religion					
Islam	89.26	9.44	1.30	0.00	0.00
Christian	89.32	10.68	0.00	0.00	0.00
Others	87.10	12.90	0.00	0.00	0.00
Ethnicity					
Jawa	94.34*	5.20	0.45	0.00	0.00
Sunda	86.00*	12.00	2.00	0.00	0.00
Sumatera	84.38*	13.28	2.34	0.00	0.00
Sulawesi	82.54*	15.87	1.59	0.00	0.00
Madura—Bali	88.46*	11.54	0.00	0.00	0.00
Others	85.96*	12.87	1.17	0.00	0.00
Monthly income (in thousand Rupiahs)					
0–500	87.38	10.87	1.75	0.00	0.00
500–2500	90.86	8.31	0.83	0.00	0.00
2500–5000	89.23	10.77	0.00	0.00	0.00
>5000	96.00	4.00	0.00	0.00	0.00

* = the proportions of self-reported health in the corresponding dimension between the demographic groups differs statistically significant, p-value <0.05

**Table 6:** EQ-5D-5L self-reported health profiles in pain/discomfort dimension in the total population sample and sub-samples by residence, gender, age, education level, religion, ethnicity and monthly income (%).

	PAIN/DISCOMFORT				
Socio-demographic	No problems	Slight Problems	Moderate problems	Severe Problems	Unable/ extreme problems
All respondents	60.32	36.55	2.56	0.57	0.00
Residence					
Rural	59.76	36.09	3.35	0.79	0.00
Urban	60.84	36.98	1.82	0.36	0.00
Gender					
Female	56.82*	39.02	3.03	1.14	0.00
Male	63.83*	34.09	2.08	0.00	0.00
Age					
17–30	63.01*	35.08	1.43	0.48	0.00
31–50	62.33*	34.70	2.28	0.68	0.00
>50	50.25*	43.72	5.53	0.50	0.00
Education					
Low	60.00	35.59	3.82	0.59	0.00
Middle	60.80	36.48	2.00	0.73	0.00
High	59.39	38.79	1.82	0.00	0.00
Religion					
Islam	60.41	36.23	2.71	0.65	0.00
Christian	63.11	34.95	1.94	0.00	0.00
Others	48.39	51.61	0.00	0.00	0.00
Ethnicity					
Jawa	67.19*	30.77	2.04	0.00	0.00
Sunda	53.50*	41.50	4.00	1.00	0.00
Sumatera	50.78*	43.75	4.69	0.78	0.00
Sulawesi	57.14*	36.51	4.76	1.59	0.00
Madura—Bali	69.23*	28.85	1.92	0.00	0.00
Others	56.14*	42.69	0.00	1.17	0.00
Monthly income (in thousand Rupiahs)					
0–500	62.33	33.59	2.91	1.17	0.00
500–2500	55.68	41.83	2.49	0.00	0.00
2500–5000	66.15	32.31	1.54	0.00	0.00
>5000	58.00	40.00	2.00	0.00	0.00

* = the proportions of self-reported health in the corresponding dimension between the demographic groups differs statistically significant, p-value <0.05

**Table 7:** EQ-5D-5L self-reported health profiles in anxiety/depression dimension in the total population sample and sub-samples by residence, gender, age, education level, religion, ethnicity and monthly income (%).

	ANXIETY/DEPRESSION				
Socio-demographic	No problems	Slight Problems	Moderate problems	Severe Problems	Unable/ extreme problems
All respondents	65.72	28.22	5.49	0.38	0.19
Residence					
Rural	67.65	26.63	5.13	0.39	0.20
Urban	63.93	29.69	5.83	0.36	0.18
Gender					
Female	62.88	30.30	6.44	0.38	0.00
Male	68.56	26.14	4.55	0.38	0.38
Age					
17–30	59.90*	30.31	8.59	0.72	0.48
31–50	70.32*	25.80	3.65	0.23	0.00
>50	67.84*	29.15	3.02	0.00	0.00
Education					
Low	65.88	27.65	6.18	0.29	0.00
Middle	65.88	28.68	4.54	0.54	0.36
High	64.85	27.88	7.27	0.00	0.00
Religion					
Islam	66.05	27.87	5.42	0.43	0.22
Christian	66.99	28.16	4.85	0.00	0.00
Others	51.61	38.71	9.68	0.00	0.00
Ethnicity					
Jawa	66.29	28.28	4.53	0.68	0.23
Sunda	67.50	24.50	7.50	0.50	0.00
Sumatera	61.72	32.03	6.25	0.00	0.00
Sulawesi	69.84	23.81	6.35	0.00	0.00
Madura—Bali	71.15	21.15	7.69	0.00	0.00
Others	61.99	33.33	4.09	0.00	0.58
Monthly income (in thousand Rupiahs)					
0–500	66.02	26.02	7.18	0.58	0.19
500–2500	62.88	33.24	3.60	0.28	0.00
2500–5000	73.08	21.54	4.62	0.00	0.77
>5000	64.00	32.00	4.00	0.00	0.00

* = the proportions of self-reported health in the corresponding dimension between the demographic groups differs statistically significant, p-value <0.05

Table 8 shows the mean EQ-VAS and index scores of the overall sample for different socio-demographic characteristics. The mean EQ-5D VAS for the overall sample was 79.39. Mean EQ-VAS scores differed between residence, age, level of education, and ethnicity groups. For instance, older respondents reported lower EQ-VAS scores than younger respondents and higher-educated respondents reported higher EQ-VAS scores than lower-educated respondents. The mean EQ-5D-5L index score was 0.911. Similar to EQ-VAS scores, gender differences were clearly observed where males had higher index scores than females. Significant differences in index scores were also reported between different age and ethnicity groups, but no clear pattern was observed.

**Table 8:** Mean scores and SD of EQ-5D-5L VAS and Index scores in the total Indonesian general population sample and sub-samples by socio-demographic characteristics.

	EQ-VAS		Index score	
	Mean	SD	Mean	SD
All respondents	79.39	14.01	0.91	0.11
Residence				
Rural	80.36*	14.15	0.91	0.11
Urban	78.49*	13.82	0.91	0.11
Gender				
Female	79.08	14.52	0.90*	0.12
Male	79.70	13.48	0.92*	0.10
Age				
17–30	80.54*	13.48	0.91*	0.11
31–50	79.42*	14.18	0.92*	0.10
>50	76.88*	14.45	0.89*	0.13
Education				
Low	76.64*	15.66	0.91	0.11
Middle	79.92*	13.30	0.91	0.11
High	83.25*	11.40	0.92	0.11
Religion				
Islam	79.54	14.00	0.91	0.11
Christian	78.81	14.17	0.92	0.11
Others	76.81	13.63	0.88	0.12
Ethnicity				
Jawa	79.37*	13.64	0.93*	0.10
Sunda	82.86*	14.29	0.91*	0.10
Sumatera	77.49*	14.44	0.89*	0.12
Sulawesi	80.84*	15.32	0.89*	0.14
Madura— Bali	79.52*	13.80	0.93*	0.11
Others	76.22*	13.02	0.89*	0.12
Monthly income**				
0–500	79.84	14.90	0.91	0.12
500–2500	78.08	13.52	0.91	0.10
2500–5000	80.79	12.01	0.93	0.09
>5000	80.50	12.19	0.91	0.11

* = the mean score between the demographic groups differs statistically significant, p-value <0.05

** = in thousand Rupiah

Details of means, standard deviations, and percentiles scores of EQ-5D-5L visual analogue scale (EQ-VAS) and index scores of the subgroups stratified by residence, gender, age, and education level could be found in the S1 Table.

### WHOQOL-BREF population norm

The EQ-5D-5L administration was accomplished in the first part of the interview, followed by the WHOQOL-BREF. Ten of the 1056 respondents of the EQ-5D-5L did not complete the WHOQOL-BREF, as they refused further involvement or because they did not have time to complete the paper questionnaire. Hence, data for the 1046 respondents was analyzed for the WHOQOL-BREF population norms. The sample mean scores for each domain, overall quality of life, and general health are presented in Table 9. There were differences in the mean quality of life scores for some sub-groups. Males reported better HRQOL in almost all domains when compared to females. Older respondents scored significantly lower on physical and social functioning. A pattern of increasing quality of life scores in all domains was observed when the level of education increased, although these differences were only statistically significant in the social and environmental domains. Regarding ethnicities, Sundanese people had the lowest mean scores in all domains whereas Maduranese and Balinese presented the highest scores in almost all domains. An income-gradient was present in almost all domains where respondents with incomes of more than 5 million Rupiah a month reported the highest quality of life.

**Table 9:** Mean scores and standard deviation (SD) of WHOQOL-BREF domains and global scores in the total population sample and sub-samples by socio-demographic characteristics.

		Overall quality of life		General health		Physical		Psychological		Social		Environmental	
	N	Mean	SD	Mean	SD	Mean	SD	Mean	SD	Mean	SD	Mean	SD
All respondents	1046	3.65	0.65	3.60	0.79	69.23	11.49	66.74	12.89	63.13	14.38	58.53	13.43
Residence													
Rural	502	3.63	0.63	3.57	0.78	68.99	11.27	67.08	12.56	62.63	13.57	58.53	13.43
Urban	544	3.66	0.67	3.64	0.80	69.45	11.70	66.43	13.19	62.63	13.57	59.25	13.74
Gender													
Female	523	3.66	0.64	3.53	0.80	68.04*	11.11	65.35*	12.46	61.82*	13.43	58.48	12.90
Male	523	3.63	0.67	3.67	0.78	70.41*	11.76	68.14*	13.17	64.44*	15.17	58.59	13.95
Age													
17–30	415	3.69*	0.66	3.65	0.81	69.82*	11.07	67.28	13.12	63.21*	14.34	59.65	13.15
31–50	434	3.65*	0.65	3.61	0.78	69.83*	11.57	66.74	13.12	64.19*	14.60	57.94	13.68
>50	197	3.55*	0.63	3.49	0.78	66.68*	11.87	65.59	11.80	60.62*	13.72	57.50	13.35
Education													
Low	334	3.44*	0.60	3.54	0.75	69.03	12.22	65.54	13.74	60.63*	14.55	56.94*	14.29
Middle	547	3.71*	0.65	3.62	0.82	69.23	11.00	67.09	12.44	63.92*	14.22	58.23*	12.70
High	165	3.85*	0.67	3.67	0.80	69.61	11.63	68.03	12.44	65.56*	13.91	62.76*	13.20
Religion													
Islam	914	3.64	0.65	3.60	0.80	69.07	11.34	66.50	12.80	63.05	14.51	58.07*	13.15
Christian	101	3.65	0.73	3.61	0.76	70.76	12.17	69.45	13.28	64.27	14.26	60.42*	15.14
Others	31	3.87	0.56	3.84	0.73	68.78	13.52	65.05	13.64	61.83	10.71	66.13*	13.33
Ethnicity													
Jawa	435	3.57*	0.66	3.61	0.73	69.78*	11.47	67.55*	12.72	62.20*	14.30	57.79*	13.13
Sunda	200	3.67*	0.62	3.46	0.87	66.48*	10.74	64.33*	11.57	61.29*	13.98	56.30*	12.16
Sumatera	127	3.68*	0.74	3.59	0.86	70.73*	11.46	68.45*	13.09	64.11*	13.91	60.46*	14.01
Sulawesi	63	3.76*	0.59	3.70	0.69	67.06*	10.53	66.01*	14.30	67.20*	15.25	61.06*	13.81
Madura—Bali	50	3.60*	0.61	3.68	0.89	74.15*	13.39	69.42*	15.78	67.17*	14.13	63.69*	16.16
Others	171	3.77*	0.61	3.71	0.81	69.28*	11.46	65.74*	12.81	64.23*	14.68	59.17*	13.56
Monthly income**													
0–500	511	3.59*	0.66	3.54*	0.81	68.59	11.97	65.75*	13.13	62.08*	13.85	57.81*	13.86
500–2.500	355	3.61*	0.63	3.61*	0.78	69.39	11.07	66.81*	13.04	62.89*	15.33	57.10*	12.74
2.500–5.000	130	3.81*	0.62	3.72*	0.77	70.11	10.89	68.57*	11.95	65.51*	12.99	61.70*	11.47
>5.000	50	4.04*	0.60	3.94*	0.68	72.36	10.55	71.75*	9.93	69.33*	14.33	67.94*	13.74

* = the mean score between the demographic groups differs statistically significant, p-value <0.05

** = in thousand Rupiah

Table 9 shows an age gradient regarding overall quality of life and general health obtained by the WHOQOL-BREF instrument: the older the respondents, the lower their overall quality of life and the more dissatisfied they were with their general health. The opposite pattern was observed for level of education and monthly income: the higher the respondents’ education and income, the better their overall quality of life and the more satisfied respondents were with their health. Details of the means, standard deviation, and percentiles scores of WHOQOL-BREF dimensions score of the subgroups stratified by residence, gender, age, and education level of this table can be found in the S2 Table.

## Discussion

This is the first study to derive norm scores for the EQ-5D-5L and WHOQOL-BREF from the Indonesian general adult population, which is the fourth most populous country in the world. We sub-divided the norm scores of the 1056 respondents according to socio-demographic characteristics, i.e. residence, gender, age, education level, income, religion, and ethnicity. We also investigated the test-retest reliability of these two instruments in 206 respondents from the original Indonesian general population sample. The EQ-5D-5L dimensions show almost perfect agreement between the two tests but poor agreement of the EQ-VAS and index scores. The WHOQOL-BREF instrument showed almost perfect agreements of the two general items and good to moderate agreement of the four domains. These findings are further discussed below.

Several limitations of this study should be considered. The respondents in our total sample mainly lived on Java island. One could therefore question the representativeness of the sample with respect to the population living over the whole archipelago. It has to be mentioned that Java is the island with the largest population of Indonesia: 57% of the population live in the island and that we also included other ethnic groups than Javanese. One way to solve this would be to interview respondents from different locations other than Java, for instance in Sumatera (west), Kalimantan (middle) and Papua (east) to determine any significant differences. Such a study could then motivate additional studies about the quality of life of people living in other parts of the archipelago.

Another limitation is that the interval time of the second test is intersect with the WHOQOL-BREF’s reference period of four weeks. This might potentially bias the test-retest result. However, this might also be considered as an advantage, since it implies that the respondent was looking partly back to a same health condition. Therefore, concerning the overlap, variation between test-retest cannot be explained by a change in the respondent’s health.

Our study found that the Indonesian EQ-5D-5L shows high agreement coefficients and percentages agreement of the five domains, but poor agreement for the EQ-VAS and the index score. The high percentage of “no problems” in the EQ-5D dimensions scores in a general population sample is common to find: e.g. South Korea [15], South Australia [33], Japan [34], and Poland [35]. The general population is usually healthy or at least has no health problem where a medical intervention or hospital admitted is needed. When no significant event that affects their health happens in the interval time of test-retest, it is encouraging that they reported similar health state in the EQ-5D-5L. On the other hand, our data has high number of respondents who reported no problems in all dimensions (health state’11111’): 44.07%. Only 33 out of 3125 (1.06%) possible health states were reported. About 80% of the test-retest respondents reported no more than one-point difference of the so-called ‘Misery index’ (i.e. sum score of the level digits) between the two tests. It can be concluded that the EQ-5D-5L data in the general population is highly skewed and shows low variance. Since ICC relies on variance, it can be expected that the ICC score is low in this population [16]. In patient data the ceiling effect is less and there is more variation in health states, hence the ICC is more favourable [17, 36–39].

The Indonesian version of WHOQOL-BREF shows good agreement of the four domains, which is consistent with previous studies in Bangladesh and Malaysia (41, 42). The two global items of the WHOQOL-BREF: overall quality of life, and general health were in almost perfect agreement. Moreover, the data points which are considered as poor agreement were less than 10% for all the domains. It can be concluded that the WHOQOL-BREF is a consistent and stable instrument to measure the quality of life of Indonesian general population.

The most self-reported health problems were observed in the pain/discomfort dimension (39.66%) and the least in the self-care dimension (1.9%). These findings were consistent with EQ-5D-5L population norm reports from other countries [15, 33–35, 40, 41]. It could be argued that self-care is a rather ‘easy’ task which is not accompanied by problems in healthy people, whilst pain/discomfort is a quite a common sign of various types of problems for which there is not one and only answer, hence respondents possibly reported problems related to pain/discomfort more often.

The mean index score of the Indonesian population was 0.91 while the mean EQ-VAS score was 79.4. The difference between index score and EQ-VAS as shown in our study is also reported by studies in South Korean (index: 0.96; EQ-VAS: 80.4) and South Australian general population (0.91; 78.6) [15, 33]. The score of WHOQOL-BREF’s domains were between 58.3 to 69.3, which is closer to the EQ-VAS score than the index score of the EQ-5D-5L. The explanation is that the top anchor of the EQ-VAS is ‘best imaginable health state’, while the best EQ-5D levels are labeled ‘no problems’. Many respondents in the general populations have a rational view on their health: although they might not experience any health problem, they are not in the best imaginable health state. For instance, a person may think that he/she is overweight, should exercise more, stop smoking, or feel a bit tired, low on energy, or have a little cold but nevertheless does not consider that a real health problem. Note that the WHOQOL-BREF also allows to estimate a value of health beyond ‘no problems’. For instance, a respondent can fill in that ‘he/she has completely enough energy for everyday life’ or ‘he/she has completely enough money to meet his/her needs’. Therefore, the EQ-VAS and WHOQOL-BREF might capture aspects in the high region of quality of life that was not captured by the five dimensions of EQ-5D reflected in the index score. To obtain estimation of quality of life in the general population, one might consider to use the WHOQOL-BREF and EQ-VAS rather than the EQ-5D-5L, as the former two might pick up variance which is not captured by the ‘no problem level’ of the EQ-5D. Note that beyond ‘no problem’, it might be in the area of ‘pleasure seeking’, instead of ‘pain avoiding’ [42], and thus should be left to private responsibility instead of collective responsibility through national health policy. However, if one would intend to use the EQ-VAS and/or index score for a sample from general population, despite of its low test-retest reliability score, it should be in a large sample size since the sample size determines the (random) error.

Similar to EQ-5D-5L results, health-related quality of life in different domains measured by WHOQOL-BREF depended on gender and age. Men had higher values in almost all domains than women. An age-gradient was present in almost all domains, especially when comparing respondents above 50 years old to those below 30. Moreover, for WHOQOL-BREF education and income influenced almost all quality of life domains, overall quality of life, and general health. The higher the respondents’ education levels and incomes, the better their quality of life and the more satisfaction with their general health. These gender, income, and education patterns were also found in studies in Denmark, Southern Brazil, and Australia, except for the age-related pattern [43–45].

Estimation of EQ-5D-5L and WHOQOL-BREF norms can contribute to the improvement of the overall health status of the Indonesian population. The population norms are important for different parties: (i) for clinicians as reference data, comparing patient data with the same demographic characteristics as in the general population, (ii) for researchers to form control groups in case series or other types of uncontrolled studies, (iii) for public health experts to assess health-related problems and to identify vulnerable groups, and (iv) for epidemiologists to determine the burden of diseases; and (v) for health care workers to determine the impact of their interventions.

## Conclusion

This study provides representative estimates of self-reported health status and quality of life for the general Indonesian population as assessed by the EQ-5D-5L and WHOQOL-BREF instruments. The descriptive system of the EQ-5D-5L and the WHOQOL-BREF have high test-retest reliability while the EQ-VAS and the index score of EQ-5D-5L show poor agreement between the tests in the general population. Our results can be useful to researchers and clinicians who can compare their findings with respect to these concepts with those of the Indonesian general population.

## Supporting information

S1 Table Population norm of the EQ-5D-5L VAS and index score stratified by subgroups. (DOCX)

S2 Table Population norm of the WHQOL-BREG domains stratified by subgroups. (DOCX)
