# Correction: Quality of life of the Indonesian general population: Test-retest reliability and population norms of the EQ-5D-5L and WHOQOL-BREF

**DOI:** 10.1371/journal.pone.0203091

**Published:** 2018-08-23

**Authors:** The PLOS ONE Staff

There is an error in the caption for Fig 1. Part of the article text was mistakenly included in the caption for Fig 1. The publisher apologies for this error.

**Fig 1:**
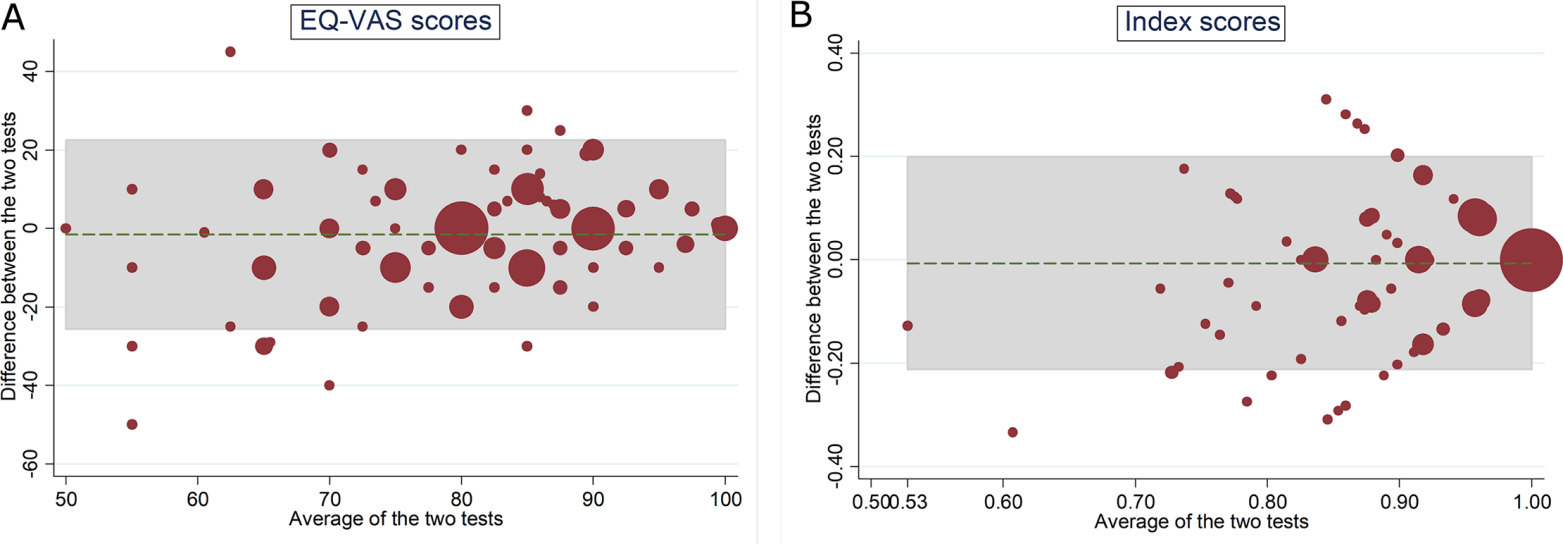
Test-retest Bland-Altman plot of the EQ-5D-5L. (A) VAS scores: 5.3% outside the limit of agreements (B) Index scores: 7.3%.

The third paragraph of the “Test-retest reliability” heading of the Results section should read as follows: Agreement coefficient (AC) of two overall items of WHOQOL-BREF: quality of life and general health, were 0.91 and 0.86, and the percentage agreement were 94.4% and 92.6%, respectively. These indicates almost perfect agreement between test and retest. ICCs of WHOQOL-BREF' four domains were between 0.70 and 0.79, which indicates moderate to good agreement (see Table 2). The Bland-Altman plot shows that the percentage of data points that lies outside the limits of agreement were 4.9% for the physical and environmental domains, 5.9% for the psychological domain, and 6.3% for the social domain. The majority of these poor agreements data points lies between mean score of 60 to 80. On the other hand, the data points in the lower part (below 60) and higher part (above 80) of the scales were all still located within the limits of agreement (see Fig 2).

Please see the complete, correct Fig 1 caption here.
