# Supplementary material for: Quality of life of the Indonesian general population: Test-retest reliability and population norms of the EQ-5D-5L and WHOQOL-BREF
Source: PLoS One. 2018 May 11;13(5):e0197098. doi: 10.1371/journal.pone.0197098 (PMC5947896; doi:10.1371/journal.pone.0197098)
Supplement: S1 Table — (DOCX) [file pone.0197098.s001.docx]

| S1 Table - Mean, standard deviation, and percentiles scores of EQ-5D-5L visual analogue scale (VAS) and utility score of the subgroups by residence, gender, age, and education level | | | | | | | | | | |
| --- | --- | --- | --- | --- | --- | --- | --- | --- | --- | --- |
| Rural, Female, 17-30 years, Low education (N=28) | | |  | Rural, Female, 17-30 years, Middle education (N=55) | | |  | Rural, Female, 17-30 years, High education (N=15) | | |
|  | VAS | Utility |  |  | VAS | Utility |  |  | VAS | Utility |
| Mean | 82.0 | 0.93 |  | Mean | 84.4 | 0.92 |  | Mean | 83.0 | 0.90 |
| SD | 17.7 | 0.10 |  | SD | 9.9 | 0.11 |  | SD | 9.8 | 0.08 |
| Perc. 5 | 50.0 | 0.78 |  | Perc. 5 | 70.0 | 0.63 |  | Perc. 5 | 70.0 | 0.75 |
| Perc. 10 | 50.0 | 0.78 |  | Perc. 10 | 70.0 | 0.77 |  | Perc. 10 | 70.0 | 0.78 |
| Perc. 25 | 70.0 | 0.91 |  | Perc. 25 | 80.0 | 0.91 |  | Perc. 25 | 75.0 | 0.84 |
| Perc. 50 | 82.5 | 0.96 |  | Perc. 50 | 90.0 | 0.92 |  | Perc. 50 | 80.0 | 0.91 |
| Perc. 75 | 100.0 | 1.00 |  | Perc. 75 | 90.0 | 1.00 |  | Perc. 75 | 91.0 | 1.00 |
| Perc .90 | 100.0 | 1.00 |  | Perc .90 | 95.0 | 1.00 |  | Perc .90 | 100.0 | 1.00 |
| Perc. 95 | 100.0 | 1.00 |  | Perc. 95 | 100.0 | 1.00 |  | Perc. 95 | 100.0 | 1.00 |
|  |  |  |  |  |  |  |  |  |  |  |
| Rural, Female, 31-50 years, Low education (N=41) | | |  | Rural, Female, 31-50 years, Middle education (N=50) | | |  | Rural, Female, 31-50 years, High education (N=15) | | |
|  | VAS | Utility |  |  | VAS | Utility |  |  | VAS | Utility |
| Mean | 83.4 | 0.92 |  | Mean | 78.3 | 0.92 |  | Mean | 87.5 | 0.96 |
| SD | 13.9 | 0.08 |  | SD | 15.8 | 0.10 |  | SD | 9.0 | 0.06 |
| Perc. 5 | 60.0 | 0.80 |  | Perc. 5 | 50.0 | 0.72 |  | Perc. 5 | 70.0 | 0.84 |
| Perc. 10 | 70.0 | 0.83 |  | Perc. 10 | 55.0 | 0.75 |  | Perc. 10 | 79.0 | 0.84 |
| Perc. 25 | 70.0 | 0.87 |  | Perc. 25 | 70.0 | 0.84 |  | Perc. 25 | 80.0 | 0.91 |
| Perc. 50 | 85.0 | 0.91 |  | Perc. 50 | 80.0 | 1.00 |  | Perc. 50 | 90.0 | 1.00 |
| Perc. 75 | 90.0 | 1.00 |  | Perc. 75 | 90.0 | 1.00 |  | Perc. 75 | 95.0 | 1.00 |
| Perc .90 | 100.0 | 1.00 |  | Perc .90 | 100.0 | 1.00 |  | Perc .90 | 100.0 | 1.00 |
| Perc. 95 | 100.0 | 1.00 |  | Perc. 95 | 100.0 | 1.00 |  | Perc. 95 | 100.0 | 1.00 |
|  |  |  |  |  |  |  |  |  |  |  |
| Rural, Female, >50 years, Low education (N=22) | | |  | Rural, Female, >50 years, Middle education (N=23) | | |  | Rural, Female, >50 years, High education (N=7) | | |
|  | VAS | Utility |  |  | VAS | Utility |  |  | VAS | Utility |
| Mean | 68.0 | 0.82 |  | Mean | 78.0 | 0.84 |  | Mean | 85.3 | 0.89 |
| SD | 16.2 | 0.17 |  | SD | 15.6 | 0.18 |  | SD | 12.2 | 0.08 |
| Perc. 5 | 50.0 | 0.53 |  | Perc. 5 | 50.0 | 0.62 |  | Perc. 5 | 70.0 | 0.80 |
| Perc. 10 | 50.0 | 0.55 |  | Perc. 10 | 60.0 | 0.62 |  | Perc. 10 | 70.0 | 0.80 |
| Perc. 25 | 50.0 | 0.78 |  | Perc. 25 | 70.0 | 0.75 |  | Perc. 25 | 70.0 | 0.84 |
| Perc. 50 | 67.5 | 0.84 |  | Perc. 50 | 80.0 | 0.91 |  | Perc. 50 | 90.0 | 0.87 |
| Perc. 75 | 75.0 | 1.00 |  | Perc. 75 | 90.0 | 1.00 |  | Perc. 75 | 97.0 | 1.00 |
| Perc .90 | 90.0 | 1.00 |  | Perc .90 | 95.0 | 1.00 |  | Perc .90 | 100.0 | 1.00 |
| Perc. 95 | 100.0 | 1.00 |  | Perc. 95 | 100.0 | 1.00 |  | Perc. 95 | 100.0 | 1.00 |
|  |  |  |  |  |  |  |  |  |  |  |
| Rural, Male, 17-30 years, Low education (N=31) | | |  | Rural, Male, 17-30 years, Middle education (N=53) | | |  | Rural, Male, 17-30 years, High education (N=16) | | |
|  | VAS | Utility |  |  | VAS | Utility |  |  | VAS | Utility |
| Mean | 78.4 | 0.94 |  | Mean | 81.1 | 0.9 |  | Mean | 83.9 | 0.91 |
| SD | 17.5 | 0.11 |  | SD | 12.5 | 0.10 |  | SD | 7.6 | 0.09 |
| Perc. 5 | 50.0 | 0.78 |  | Perc. 5 | 51.0 | 0.69 |  | Perc. 5 | 70.0 | 0.72 |
| Perc. 10 | 60.0 | 0.84 |  | Perc. 10 | 70.0 | 0.83 |  | Perc. 10 | 71.0 | 0.78 |
| Perc. 25 | 70.0 | 0.91 |  | Perc. 25 | 70.0 | 0.84 |  | Perc. 25 | 80.0 | 0.84 |
| Perc. 50 | 80.0 | 1.00 |  | Perc. 50 | 80.0 | 0.91 |  | Perc. 50 | 82.5 | 0.92 |
| Perc. 75 | 90.0 | 1.00 |  | Perc. 75 | 90.0 | 1.00 |  | Perc. 75 | 90.0 | 1.00 |
| Perc .90 | 100.0 | 1.00 |  | Perc .90 | 100.0 | 1.00 |  | Perc .90 | 95.0 | 1.00 |
| Perc. 95 | 100.0 | 1.00 |  | Perc. 95 | 100.0 | 1.00 |  | Perc. 95 | 96.0 | 1.00 |
|  |  |  |  |  |  |  |  |  |  |  |
| Rural, Male, 31-50 years, Low education (N=40) | | |  | Rural, Male, 31-50 years, Middle education (N=50) | | |  | Rural, Male, 31-50 years, High education (N=15) | | |
|  | VAS | Utility |  |  | VAS | Utility |  |  | VAS | Utility |
| Mean | 75.6 | 0.96 |  | Mean | 80.7 | 0.91 |  | Mean | 87.7 | 0.98 |
| SD | 16.5 | 0.07 |  | SD | 12.6 | 0.10 |  | SD | 9.2 | 0.05 |
| Perc. 5 | 47.5 | 0.78 |  | Perc. 5 | 60.0 | 0.72 |  | Perc. 5 | 70.0 | 0.84 |
| Perc. 10 | 55.0 | 0.87 |  | Perc. 10 | 67.5 | 0.83 |  | Perc. 10 | 80.0 | 0.91 |
| Perc. 25 | 70.0 | 0.96 |  | Perc. 25 | 70.0 | 0.84 |  | Perc. 25 | 80.0 | 1.00 |
| Perc. 50 | 70.0 | 1.00 |  | Perc. 50 | 80.0 | 0.92 |  | Perc. 50 | 90.0 | 1.00 |
| Perc. 75 | 90.0 | 1.00 |  | Perc. 75 | 90.0 | 1.00 |  | Perc. 75 | 95.0 | 1.00 |
| Perc .90 | 100.0 | 1.00 |  | Perc .90 | 100.0 | 1.00 |  | Perc .90 | 100.0 | 1.00 |
| Perc. 95 | 100.0 | 1.00 |  | Perc. 95 | 100.0 | 1.00 |  | Perc. 95 | 100.0 | 1.00 |
|  |  |  |  |  |  |  |  |  |  |  |
| Rural, Male, >50 years, Low education (N=22) | | |  | Rural, Male, >50 years, Middle education (N=17) | | |  | Rural, Male, >50 years, High education (N=7) | | |
|  | VAS | Utility |  |  | VAS | Utility |  |  | VAS | Utility |
| Mean | 78.4 | 0.91 |  | Mean | 75.7 | 0.89 |  | Mean | 81.4 | 0.89 |
| SD | 11.4 | 0.09 |  | SD | 15.3 | 0.11 |  | SD | 10.3 | 0.13 |
| Perc. 5 | 60.0 | 0.75 |  | Perc. 5 | 50.0 | 0.62 |  | Perc. 5 | 70.0 | 0.64 |
| Perc. 10 | 65.0 | 0.78 |  | Perc. 10 | 50.0 | 0.63 |  | Perc. 10 | 70.0 | 0.64 |
| Perc. 25 | 70.0 | 0.84 |  | Perc. 25 | 70.0 | 0.84 |  | Perc. 25 | 70.0 | 0.83 |
| Perc. 50 | 80.0 | 0.91 |  | Perc. 50 | 80.0 | 0.91 |  | Perc. 50 | 80.0 | 0.91 |
| Perc. 75 | 90.0 | 1.00 |  | Perc. 75 | 90.0 | 1.00 |  | Perc. 75 | 95.0 | 1.00 |
| Perc .90 | 90.0 | 1.00 |  | Perc .90 | 90.0 | 1.00 |  | Perc .90 | 95.0 | 1.00 |
| Perc. 95 | 90.0 | 1.00 |  | Perc. 95 | 100.0 | 1.00 |  | Perc. 95 | 95.0 | 1.00 |
|  | | | | | | | | | | |
| Urban, Female, 17-30 years, Low education (N=33) | | |  | Urban, Female, 17-30 years, Middle education (N=57) | | |  | Urban, Female, 17-30 years, High education (N=18) | | |
|  | VAS | Utility |  |  | VAS | Utility |  |  | VAS | Utility |
| Mean | 73.2 | 0.91 |  | Mean | 79.2 | 0.91 |  | Mean | 80.5 | 0.87 |
| SD | 11.6 | 0.08 |  | SD | 14.1 | 0.09 |  | SD | 15.5 | 0.12 |
| Perc. 5 | 50.0 | 0.82 |  | Perc. 5 | 50.0 | 0.75 |  | Perc. 5 | 40.0 | 0.63 |
| Perc. 10 | 60.0 | 0.84 |  | Perc. 10 | 60.0 | 0.78 |  | Perc. 10 | 60.0 | 0.67 |
| Perc. 25 | 70.0 | 0.84 |  | Perc. 25 | 70.0 | 0.84 |  | Perc. 25 | 75.0 | 0.78 |
| Perc. 50 | 70.0 | 0.92 |  | Perc. 50 | 80.0 | 0.92 |  | Perc. 50 | 80.0 | 0.91 |
| Perc. 75 | 80.0 | 1.00 |  | Perc. 75 | 90.0 | 1.00 |  | Perc. 75 | 92.0 | 1.00 |
| Perc .90 | 90.0 | 1.00 |  | Perc .90 | 95.0 | 1.00 |  | Perc .90 | 98.0 | 1.00 |
| Perc. 95 | 90.0 | 1.00 |  | Perc. 95 | 100.0 | 1.00 |  | Perc. 95 | 100.0 | 1.00 |
|  |  |  |  |  |  |  |  |  |  |  |
| Urban, Female, 31-50 years, Low education (N=32) | | |  | Urban, Female, 31-50 years, Middle education (N=62) | | |  | Urban, Female, 31-50 years, High education (N=18) | | |
|  | VAS | Utility |  |  | VAS | Utility |  |  | VAS | Utility |
| Mean | 74.8 | 0.85 |  | Mean | 75.3 | 0.89 |  | Mean | 86.7 | 0.89 |
| SD | 17.8 | 0.14 |  | SD | 13.8 | 0.13 |  | SD | 8.0 | 0.13 |
| Perc. 5 | 30.0 | 0.52 |  | Perc. 5 | 50.0 | 0.71 |  | Perc. 5 | 75.0 | 0.69 |
| Perc. 10 | 57.0 | 0.72 |  | Perc. 10 | 60.0 | 0.72 |  | Perc. 10 | 75.0 | 0.84 |
| Perc. 25 | 67.0 | 0.80 |  | Perc. 25 | 70.0 | 0.84 |  | Perc. 25 | 80.0 | 0.91 |
| Perc. 50 | 70.0 | 0.85 |  | Perc. 50 | 80.0 | 0.91 |  | Perc. 50 | 90.0 | 1.00 |
| Perc. 75 | 90.0 | 1.00 |  | Perc. 75 | 80.0 | 1.00 |  | Perc. 75 | 90.0 | 1.00 |
| Perc .90 | 100.0 | 1.00 |  | Perc .90 | 90.0 | 1.00 |  | Perc .90 | 100.0 | 1.00 |
| Perc. 95 | 100.0 | 1.00 |  | Perc. 95 | 90.0 | 1.00 |  | Perc. 95 | 100.0 | 1.00 |
|  |  |  |  |  |  |  |  |  |  |  |
| Urban, Female, >50 years, Low education (N=20) | | |  | Urban, Female, >50 years, Middle education (N=25) | | |  | Urban, Female, >50 years, High education (N=7) | | |
|  | VAS | Utility |  |  | VAS | Utility |  |  | VAS | Utility |
| Mean | 75.9 | 0.85 |  | Mean | 78.4 | 0.89 |  | Mean | 80.0 | 0.92 |
| SD | 13.2 | 0.13 |  | SD | 16.5 | 0.13 |  | SD | 9.6 | 0.08 |
| Perc. 5 | 55.0 | 0.58 |  | Perc. 5 | 50.0 | 0.63 |  | Perc. 5 | 65.0 | 0.80 |
| Perc. 10 | 60.0 | 0.65 |  | Perc. 10 | 50.0 | 0.69 |  | Perc. 10 | 65.0 | 0.80 |
| Perc. 25 | 69.0 | 0.80 |  | Perc. 25 | 70.0 | 0.80 |  | Perc. 25 | 70.0 | 0.84 |
| Perc. 50 | 80.0 | 0.87 |  | Perc. 50 | 80.0 | 0.92 |  | Perc. 50 | 80.0 | 0.92 |
| Perc. 75 | 80.0 | 0.92 |  | Perc. 75 | 90.0 | 1.00 |  | Perc. 75 | 90.0 | 1.00 |
| Perc .90 | 95.0 | 1.00 |  | Perc .90 | 100.0 | 1.00 |  | Perc .90 | 90.0 | 1.00 |
| Perc. 95 | 100.0 | 1.00 |  | Perc. 95 | 100.0 | 1.00 |  | Perc. 95 | 90.0 | 1.00 |
|  |  |  |  |  |  |  |  |  |  |  |
| Urban, Male, 17-30 years, Low education (N=24) | | |  | Urban, Male, 17-30 years, Middle education (N=66) | | |  | Urban, Male, 17-30 years, High education (N=23) | | |
|  | VAS | Utility |  |  | VAS | Utility |  |  | VAS | Utility |
| Mean | 75.3 | 0.93 |  | Mean | 82.8 | 0.92 |  | Mean | 80.3 | 0.86 |
| SD | 13.6 | 0.08 |  | SD | 12.2 | 0.11 |  | SD | 15.2 | 0.17 |
| Perc. 5 | 50.0 | 0.78 |  | Perc. 5 | 60.0 | 0.69 |  | Perc. 5 | 55.0 | 0.71 |
| Perc. 10 | 60.0 | 0.84 |  | Perc. 10 | 70.0 | 0.78 |  | Perc. 10 | 70.0 | 0.71 |
| Perc. 25 | 65.0 | 0.87 |  | Perc. 25 | 80.0 | 0.87 |  | Perc. 25 | 70.0 | 0.84 |
| Perc. 50 | 79.5 | 0.92 |  | Perc. 50 | 85.0 | 0.92 |  | Perc. 50 | 80.0 | 0.91 |
| Perc. 75 | 80.0 | 1.00 |  | Perc. 75 | 90.0 | 1.00 |  | Perc. 75 | 95.0 | 0.92 |
| Perc .90 | 90.0 | 1.00 |  | Perc .90 | 95.0 | 1.00 |  | Perc .90 | 98.0 | 1.00 |
| Perc. 95 | 100.0 | 1.00 |  | Perc. 95 | 100.0 | 1.00 |  | Perc. 95 | 100.0 | 1.00 |
|  |  |  |  |  |  |  |  |  |  |  |
| Urban, Male, 31-50 years, Low education (N=31) | | |  | Urban, Male, 31-50 years, Middle education (N=67) | | |  | Urban, Male, 31-50 years, High education (N= 17) | | |
|  | VAS | Utility |  |  | VAS | Utility |  |  | VAS | Utility |
| Mean | 78.4 | 0.95 |  | Mean | 79.9 | 0.93 |  | Mean | 80.0 | 0.93 |
| SD | 16.1 | 0.05 |  | SD | 11.8 | 0.08 |  | SD | 9.7 | 0.08 |
| Perc. 5 | 50.0 | 0.87 |  | Perc. 5 | 60.0 | 0.80 |  | Perc. 5 | 70.0 | 0.82 |
| Perc. 10 | 50.0 | 0.91 |  | Perc. 10 | 65.0 | 0.84 |  | Perc. 10 | 70.0 | 0.83 |
| Perc. 25 | 70.0 | 0.91 |  | Perc. 25 | 70.0 | 0.84 |  | Perc. 25 | 70.0 | 0.84 |
| Perc. 50 | 80.0 | 1.00 |  | Perc. 50 | 80.0 | 1.00 |  | Perc. 50 | 80.0 | 0.92 |
| Perc. 75 | 90.0 | 1.00 |  | Perc. 75 | 90.0 | 1.00 |  | Perc. 75 | 90.0 | 1.00 |
| Perc .90 | 95.0 | 1.00 |  | Perc .90 | 95.0 | 1.00 |  | Perc .90 | 95.0 | 1.00 |
| Perc. 95 | 100.0 | 1.00 |  | Perc. 95 | 95.0 | 1.00 |  | Perc. 95 | 95.0 | 1.00 |
|  |  |  |  |  |  |  |  |  |  |  |
| Urban, Male, >50 years, Low education (N=16) | | |  | Urban, Male, >50 years, Middle education (N=26) | | |  | Urban, Male, >50 years, High education (N=7) | | |
|  | VAS | Utility |  |  | VAS | Utility |  |  | VAS | Utility |
| Mean | 69.2 | 0.87 |  | Mean | 81.0 | 0.95 |  | Mean | 82.9 | 0.97 |
| SD | 14.8 | 0.12 |  | SD | 11.1 | 0.09 |  | SD | 16.0 | 0.05 |
| Perc. 5 | 40.0 | 0.53 |  | Perc. 5 | 65.0 | 0.72 |  | Perc. 5 | 50.0 | 0.88 |
| Perc. 10 | 50.0 | 0.74 |  | Perc. 10 | 70.0 | 0.80 |  | Perc. 10 | 50.0 | 0.88 |
| Perc. 25 | 60.0 | 0.83 |  | Perc. 25 | 70.0 | 0.92 |  | Perc. 25 | 80.0 | 0.92 |
| Perc. 50 | 70.0 | 0.86 |  | Perc. 50 | 80.0 | 1.00 |  | Perc. 50 | 90.0 | 1.00 |
| Perc. 75 | 80.0 | 0.96 |  | Perc. 75 | 90.0 | 1.00 |  | Perc. 75 | 90.0 | 1.00 |
| Perc .90 | 90.0 | 1.00 |  | Perc .90 | 100.0 | 1.00 |  | Perc .90 | 100.0 | 1.00 |
| Perc. 95 | 100.0 | 1.00 |  | Perc. 95 | 100.0 | 1.00 |  | Perc. 95 | 100.0 | 1.00 |
| Perc: Percentile  *: Low education means primary school and below, middle education means high school, and high education is college/university | | | | | | | | | | |
