# Supplementary material for: Quality of life of the Indonesian general population: Test-retest reliability and population norms of the EQ-5D-5L and WHOQOL-BREF
Source: PLoS One. 2018 May 11;13(5):e0197098. doi: 10.1371/journal.pone.0197098 (PMC5947896; doi:10.1371/journal.pone.0197098)
Supplement: S2 Table — (DOCX) [file pone.0197098.s002.docx]

| S2 Table - Mean, standard deviation, and percentiles scores of WHOQOL-BREF dimensions score of the subgroups by residence, gender, age, and education level | | | | | | | | | | |
| --- | --- | --- | --- | --- | --- | --- | --- | --- | --- | --- |
| Rural, Female, 17-30 years, Low education* (N=28) | | | | |  | Rural, Female, 17-30 years, Middle education (N=55) | | | | |
|  | Physical | Psychological | Social | Environment |  |  | Physical | Psychological | Social | Environment |
| Mean | 66.2 | 67.7 | 60.4 | 57.9 |  | Mean | 69.4 | 65.4 | 59.7 | 56.9 |
| SD | 10.0 | 11.9 | 14.5 | 14.1 |  | SD | 8.9 | 11.8 | 11.2 | 11.7 |
| Perc. 5 | 50.0 | 50.0 | 41.7 | 40.6 |  | Perc. 5 | 57.1 | 41.7 | 41.7 | 34.4 |
| Perc. 10 | 53.6 | 50.0 | 50.0 | 46.9 |  | Perc. 10 | 57.1 | 50.0 | 41.7 | 43.8 |
| Perc. 25 | 58.9 | 60.4 | 50.0 | 53.1 |  | Perc. 25 | 64.3 | 58.3 | 50.0 | 50.0 |
| Perc. 50 | 64.3 | 66.7 | 58.3 | 59.4 |  | Perc. 50 | 67.9 | 66.7 | 58.3 | 56.3 |
| Perc. 75 | 71.4 | 75.0 | 75.0 | 65.6 |  | Perc. 75 | 75.0 | 70.8 | 66.7 | 65.6 |
| Perc .90 | 78.6 | 83.3 | 83.3 | 78.1 |  | Perc .90 | 82.1 | 79.2 | 75.0 | 75.0 |
| Perc. 95 | 85.7 | 83.3 | 83.3 | 84.4 |  | Perc. 95 | 82.1 | 87.5 | 75.0 | 75.0 |
|  |  |  |  |  |  |  |  |  |  |  |
| Rural, Female, 17-30 years, High education (N=15) | | | | |  | Rural, Female, 31-50 years, Low education (N=41) | | | | |
|  | Physical | Psychological | Social | Environment |  |  | Physical | Psychological | Social | Environment |
| Mean | 69.3 | 68.1 | 68.9 | 67.5 |  | Mean | 66.8 | 63.2 | 63.0 | 54.4 |
| SD | 8.1 | 14.1 | 12.0 | 9.1 |  | SD | 12.6 | 13.6 | 14.8 | 13.2 |
| Perc. 5 | 53.6 | 50.0 | 50.0 | 56.3 |  | Perc. 5 | 50.0 | 45.8 | 33.3 | 31.3 |
| Perc. 10 | 57.1 | 50.0 | 50.0 | 56.3 |  | Perc. 10 | 53.6 | 50.0 | 50.0 | 34.4 |
| Perc. 25 | 64.3 | 54.2 | 66.7 | 59.4 |  | Perc. 25 | 60.7 | 54.2 | 50.0 | 43.8 |
| Perc. 50 | 71.4 | 66.7 | 66.7 | 68.8 |  | Perc. 50 | 64.3 | 58.3 | 66.7 | 53.1 |
| Perc. 75 | 78.6 | 79.2 | 75.0 | 71.9 |  | Perc. 75 | 71.4 | 66.7 | 75.0 | 62.5 |
| Perc .90 | 78.6 | 87.5 | 91.7 | 75.0 |  | Perc .90 | 82.1 | 79.2 | 75.0 | 68.8 |
| Perc. 95 | 78.6 | 91.7 | 91.7 | 90.6 |  | Perc. 95 | 85.7 | 87.5 | 83.3 | 71.9 |
|  |  |  |  |  |  |  |  |  |  |  |
| Rural, Female, 31-50 years, Middle education (N=50) | | | | |  | Rural, Female, 31-50 years, High education (N=15) | | | | |
|  | Physical | Psychological | Social | Environment |  |  | Physical | Psychological | Social | Environment |
| Mean | 67.9 | 65.8 | 63.1 | 57.4 |  | Mean | 71.9 | 71.4 | 68.2 | 64.2 |
| SD | 11.3 | 12.9 | 11.2 | 10.7 |  | SD | 9.3 | 7.0 | 9.7 | 6.9 |
| Perc. 5 | 50.0 | 45.8 | 50.0 | 37.5 |  | Perc. 5 | 60.7 | 58.3 | 50.0 | 53.1 |
| Perc. 10 | 53.6 | 50.0 | 50.0 | 46.9 |  | Perc. 10 | 60.7 | 58.3 | 50.0 | 53.1 |
| Perc. 25 | 60.7 | 58.3 | 50.0 | 50.0 |  | Perc. 25 | 60.7 | 66.7 | 58.3 | 59.4 |
| Perc. 50 | 67.9 | 66.7 | 58.3 | 56.3 |  | Perc. 50 | 71.4 | 70.8 | 75.0 | 65.6 |
| Perc. 75 | 75.0 | 75.0 | 75.0 | 62.5 |  | Perc. 75 | 78.6 | 75.0 | 75.0 | 68.8 |
| Perc .90 | 78.6 | 79.2 | 75.0 | 73.4 |  | Perc .90 | 82.1 | 79.2 | 75.0 | 75.0 |
| Perc. 95 | 89.3 | 91.7 | 75.0 | 75.0 |  | Perc. 95 | 89.3 | 83.3 | 83.3 | 75.0 |
|  |  |  |  |  |  |  |  |  |  |  |
| Rural, Female, >50 years, Low education (N=22) | | | | |  | Rural, Female, >50 years, Middle education (N=23) | | | | |
|  | Physical | Psychological | Social | Environment |  |  | Physical | Psychological | Social | Environment |
| Mean | 67.7 | 67.4 | 56.9 | 54.5 |  | Mean | 66.6 | 64.5 | 59.4 | 62.2 |
| SD | 11.0 | 12.6 | 19.2 | 17.9 |  | SD | 12.5 | 11.5 | 11.3 | 11.8 |
| Perc. 5 | 50.0 | 50.0 | 25.0 | 37.5 |  | Perc. 5 | 46.4 | 41.7 | 50.0 | 43.8 |
| Perc. 10 | 57.1 | 54.2 | 25.0 | 37.5 |  | Perc. 10 | 50.0 | 50.0 | 50.0 | 46.9 |
| Perc. 25 | 64.3 | 66.7 | 50.0 | 46.9 |  | Perc. 25 | 57.1 | 58.3 | 50.0 | 56.3 |
| Perc. 50 | 67.9 | 66.7 | 58.3 | 53.1 |  | Perc. 50 | 67.9 | 66.7 | 58.3 | 60.9 |
| Perc. 75 | 78.6 | 75.0 | 66.7 | 65.6 |  | Perc. 75 | 75.0 | 70.8 | 66.7 | 68.8 |
| Perc .90 | 85.7 | 79.2 | 75.0 | 75.0 |  | Perc .90 | 82.1 | 75.0 | 75.0 | 78.1 |
| Perc. 95 | 85.7 | 95.8 | 83.3 | 81.3 |  | Perc. 95 | 85.7 | 75.0 | 75.0 | 81.3 |
|  |  |  |  |  |  |  |  |  |  |  |
| Rural, Female, >50 years, High education (N=7) | | | | |  | Rural, Male, 17-30 years, Low education (N=31) | | | | |
|  | Physical | Psychological | Social | Environment |  |  | Physical | Psychological | Social | Environment |
| Mean | 64.3 | 69.4 | 63.9 | 56.8 |  | Mean | 71.8 | 66.4 | 59.7 | 55.3 |
| SD | 9.3 | 10.4 | 11.4 | 13.2 |  | SD | 11.8 | 14.1 | 12.6 | 15.0 |
| Perc. 5 | 50.0 | 54.2 | 50.0 | 37.5 |  | Perc. 5 | 50.0 | 50.0 | 50.0 | 40.6 |
| Perc. 10 | 50.0 | 54.2 | 50.0 | 37.5 |  | Perc. 10 | 57.1 | 50.0 | 50.0 | 40.6 |
| Perc. 25 | 60.7 | 62.5 | 50.0 | 50.0 |  | Perc. 25 | 64.3 | 54.2 | 50.0 | 43.8 |
| Perc. 50 | 64.3 | 75.0 | 66.7 | 62.5 |  | Perc. 50 | 71.4 | 64.6 | 54.2 | 53.1 |
| Perc. 75 | 78.6 | 79.2 | 75.0 | 68.8 |  | Perc. 75 | 78.6 | 79.2 | 75.0 | 65.6 |
| Perc .90 | 82.1 | 83.3 | 75.0 | 71.9 |  | Perc .90 | 87.5 | 89.6 | 75.0 | 82.8 |
| Perc. 95 | 82.1 | 83.3 | 75.0 | 71.9 |  | Perc. 95 | 92.9 | 91.7 | 83.3 | 84.4 |
|  |  |  |  |  |  |  |  |  |  |  |
| Rural, Male, 17-30 years, Middle education (N=53) | | | | |  | Rural, Male, 17-30 years, High education (N=16) | | | | |
|  | Physical | Psychological | Social | Environment |  |  | Physical | Psychological | Social | Environment |
| Mean | 72.0 | 70.3 | 67.9 | 60.2 |  | Mean | 70.1 | 71.4 | 67.7 | 65.2 |
| SD | 9.5 | 13.3 | 14.1 | 12.9 |  | SD | 9.8 | 12.6 | 11.3 | 13.7 |
| Perc. 5 | 53.6 | 45.8 | 41.7 | 37.5 |  | Perc. 5 | 46.4 | 37.5 | 33.3 | 31.3 |
| Perc. 10 | 57.1 | 54.2 | 50.0 | 43.8 |  | Perc. 10 | 60.7 | 54.2 | 58.3 | 50.0 |
| Perc. 25 | 67.9 | 62.5 | 58.3 | 50.0 |  | Perc. 25 | 66.1 | 68.8 | 66.7 | 62.5 |
| Perc. 50 | 75.0 | 70.8 | 66.7 | 59.4 |  | Perc. 50 | 69.6 | 72.9 | 66.7 | 65.6 |
| Perc. 75 | 78.6 | 79.2 | 75.0 | 68.8 |  | Perc. 75 | 76.8 | 79.2 | 75.0 | 73.4 |
| Perc .90 | 82.1 | 87.5 | 83.3 | 75.0 |  | Perc .90 | 82.1 | 83.3 | 75.0 | 81.3 |
| Perc. 95 | 85.7 | 91.7 | 91.7 | 81.3 |  | Perc. 95 | 85.7 | 87.5 | 83.3 | 87.5 |
|  |  |  |  |  |  |  |  |  |  |  |
| Rural, Male, 31-50 years, Low education (N=40) | | | | |  | Rural, Male, 31-50 years, Middle education (N=50) | | | | |
|  | Physical | Psychological | Social | Environment |  |  | Physical | Psychological | Social | Environment |
| Mean | 73.4 | 68.5 | 60.7 | 57.4 |  | Mean | 68.6 | 67.2 | 62.4 | 54.0 |
| SD | 12.3 | 13.1 | 14.7 | 14.2 |  | SD | 11.2 | 10.5 | 12.2 | 11.2 |
| Perc. 5 | 50.0 | 50.0 | 41.7 | 37.5 |  | Perc. 5 | 50.0 | 54.2 | 50.0 | 37.5 |
| Perc. 10 | 57.1 | 50.0 | 41.7 | 40.6 |  | Perc. 10 | 53.6 | 56.3 | 50.0 | 40.6 |
| Perc. 25 | 67.9 | 54.2 | 50.0 | 43.8 |  | Perc. 25 | 64.3 | 58.3 | 50.0 | 46.9 |
| Perc. 50 | 75.0 | 66.7 | 58.3 | 59.4 |  | Perc. 50 | 71.4 | 66.7 | 62.5 | 51.6 |
| Perc. 75 | 82.1 | 79.2 | 75.0 | 65.6 |  | Perc. 75 | 78.6 | 75.0 | 75.0 | 62.5 |
| Perc .90 | 89.3 | 83.3 | 75.0 | 78.1 |  | Perc .90 | 80.4 | 81.3 | 75.0 | 71.9 |
| Perc. 95 | 92.9 | 87.5 | 83.3 | 84.4 |  | Perc. 95 | 82.1 | 87.5 | 75.0 | 75.0 |
|  |  |  |  |  |  |  |  |  |  |  |
| Rural, Male, 31-50 years, High education (N=15) | | | | |  | Rural, Male, >50 years, Low education (N=22) | | | | |
|  | Physical | Psychological | Social | Environment |  |  | Physical | Psychological | Social | Environment |
| Mean | 75.9 | 75.3 | 71.9 | 66.8 |  | Mean | 61.6 | 59.0 | 53.3 | 49.2 |
| SD | 12.4 | 13.1 | 16.6 | 12.5 |  | SD | 11.0 | 11.5 | 11.0 | 14.9 |
| Perc. 5 | 50.0 | 54.2 | 50.0 | 50.0 |  | Perc. 5 | 50.0 | 50.0 | 41.7 | 28.1 |
| Perc. 10 | 60.7 | 58.3 | 50.0 | 53.1 |  | Perc. 10 | 50.0 | 50.0 | 41.7 | 37.5 |
| Perc. 25 | 67.9 | 66.7 | 58.3 | 56.3 |  | Perc. 25 | 50.0 | 50.0 | 50.0 | 40.6 |
| Perc. 50 | 75.0 | 75.0 | 66.7 | 65.6 |  | Perc. 50 | 60.7 | 58.3 | 50.0 | 48.4 |
| Perc. 75 | 85.7 | 83.3 | 83.3 | 78.1 |  | Perc. 75 | 71.4 | 66.7 | 58.3 | 62.5 |
| Perc .90 | 92.9 | 95.8 | 100.0 | 87.5 |  | Perc .90 | 75.0 | 75.0 | 75.0 | 65.6 |
| Perc. 95 | 96.4 | 100.0 | 100.0 | 90.6 |  | Perc. 95 | 78.6 | 79.2 | 75.0 | 65.6 |
|  |  |  |  |  |  |  |  |  |  |  |
| Rural, Male, >50 years, Middle education (N=17) | | | | |  | Rural, Male, >50 years, High education (N=7) | | | | |
|  | Physical | Psychological | Social | Environment |  |  | Physical | Psychological | Social | Environment |
| Mean | 63.2 | 68.8 | 66.7 | 56.5 |  | Mean | 64.9 | 67.4 | 56.9 | 57.8 |
| SD | 14.0 | 12.3 | 15.2 | 9.0 |  | SD | 13.3 | 9.7 | 6.3 | 6.2 |
| Perc. 5 | 39.3 | 41.7 | 41.7 | 37.5 |  | Perc. 5 | 42.9 | 54.2 | 50.0 | 50.0 |
| Perc. 10 | 42.9 | 50.0 | 50.0 | 43.8 |  | Perc. 10 | 42.9 | 54.2 | 50.0 | 50.0 |
| Perc. 25 | 50.0 | 62.5 | 58.3 | 53.1 |  | Perc. 25 | 60.7 | 62.5 | 50.0 | 50.0 |
| Perc. 50 | 71.4 | 70.8 | 66.7 | 56.3 |  | Perc. 50 | 64.3 | 66.7 | 58.3 | 59.4 |
| Perc. 75 | 75.0 | 79.2 | 75.0 | 62.5 |  | Perc. 75 | 78.6 | 70.8 | 66.7 | 62.5 |
| Perc .90 | 78.6 | 79.2 | 83.3 | 68.8 |  | Perc .90 | 78.6 | 83.3 | 66.7 | 62.5 |
| Perc. 95 | 78.6 | 83.3 | 100.0 | 71.9 |  | Perc. 95 | 78.6 | 83.3 | 66.7 | 62.5 |
|  |  |  |  |  |  |  |  |  |  |  |
| Urban, Female, 17-30 years, Low education (N=33) | | | | |  | Urban, Female, 17-30 years, Middle education (N=57) | | | | |
|  | Physical | Psychological | Social | Environment |  |  | Physical | Psychological | Social | Environment |
| Mean | 68.7 | 63.7 | 57.5 | 57.5 |  | Mean | 68.3 | 65.5 | 62.2 | 59.4 |
| SD | 11.1 | 14.7 | 10.6 | 12.3 |  | SD | 11.0 | 13.2 | 16.5 | 13.8 |
| Perc. 5 | 50.0 | 37.5 | 50.0 | 34.4 |  | Perc. 5 | 53.6 | 45.8 | 33.3 | 40.6 |
| Perc. 10 | 53.6 | 50.0 | 50.0 | 43.8 |  | Perc. 10 | 53.6 | 54.2 | 41.7 | 43.8 |
| Perc. 25 | 60.7 | 54.2 | 50.0 | 46.9 |  | Perc. 25 | 60.7 | 58.3 | 50.0 | 53.1 |
| Perc. 50 | 71.4 | 62.5 | 50.0 | 59.4 |  | Perc. 50 | 67.9 | 66.7 | 58.3 | 59.4 |
| Perc. 75 | 78.6 | 66.7 | 66.7 | 62.5 |  | Perc. 75 | 75.0 | 70.8 | 75.0 | 65.6 |
| Perc .90 | 78.6 | 83.3 | 75.0 | 71.9 |  | Perc .90 | 82.1 | 83.3 | 83.3 | 75.0 |
| Perc. 95 | 85.7 | 91.7 | 75.0 | 81.3 |  | Perc. 95 | 89.3 | 91.7 | 91.7 | 93.8 |
|  |  |  |  |  |  |  |  |  |  |  |
| Urban, Female, 17-30 years, High education (N=18) | | | | |  | Urban, Female, 31-50 years, Low education (N=32) | | | | |
|  | Physical | Psychological | Social | Environment |  |  | Physical | Psychological | Social | Environment |
| Mean | 65.9 | 65.4 | 61.3 | 60.8 |  | Mean | 66.8 | 59.7 | 59.6 | 53.4 |
| SD | 14.6 | 10.7 | 15.1 | 15.6 |  | SD | 12.0 | 12.0 | 13.5 | 10.6 |
| Perc. 5 | 35.7 | 37.5 | 33.3 | 34.4 |  | Perc. 5 | 42.9 | 41.7 | 33.3 | 37.5 |
| Perc. 10 | 39.3 | 54.2 | 41.7 | 43.8 |  | Perc. 10 | 50.0 | 45.8 | 41.7 | 43.8 |
| Perc. 25 | 57.1 | 62.5 | 50.0 | 46.9 |  | Perc. 25 | 58.9 | 50.0 | 50.0 | 46.9 |
| Perc. 50 | 67.9 | 66.7 | 62.5 | 62.5 |  | Perc. 50 | 67.9 | 58.3 | 58.3 | 53.1 |
| Perc. 75 | 78.6 | 70.8 | 66.7 | 71.9 |  | Perc. 75 | 75.0 | 66.7 | 66.7 | 59.4 |
| Perc .90 | 85.7 | 70.8 | 83.3 | 75.0 |  | Perc .90 | 78.6 | 75.0 | 75.0 | 68.8 |
| Perc. 95 | 85.7 | 83.3 | 91.7 | 84.4 |  | Perc. 95 | 82.1 | 79.2 | 75.0 | 71.9 |
|  |  |  |  |  |  |  |  |  |  |  |
| Urban, Female, 31-50 years, Middle education (N=62) | | | | |  | Urban, Female, 31-50 years, High education (N=18) | | | | |
|  | Physical | Psychological | Social | Environment |  |  | Physical | Psychological | Social | Environment |
| Mean | 69.8 | 66.3 | 64.5 | 58.8 |  | Mean | 73.0 | 68.2 | 66.2 | 66.2 |
| SD | 10.4 | 12.6 | 13.1 | 13.9 |  | SD | 12.0 | 9.2 | 13.4 | 12.8 |
| Perc. 5 | 53.6 | 45.8 | 50.0 | 34.4 |  | Perc. 5 | 42.9 | 54.2 | 41.7 | 40.6 |
| Perc. 10 | 57.1 | 50.0 | 50.0 | 43.8 |  | Perc. 10 | 57.1 | 54.2 | 50.0 | 43.8 |
| Perc. 25 | 60.7 | 54.2 | 50.0 | 50.0 |  | Perc. 25 | 67.9 | 58.3 | 50.0 | 56.3 |
| Perc. 50 | 67.9 | 66.7 | 66.7 | 56.3 |  | Perc. 50 | 71.4 | 70.8 | 75.0 | 67.2 |
| Perc. 75 | 75.0 | 75.0 | 75.0 | 65.6 |  | Perc. 75 | 82.1 | 75.0 | 75.0 | 78.1 |
| Perc .90 | 82.1 | 79.2 | 75.0 | 78.1 |  | Perc .90 | 89.3 | 83.3 | 83.3 | 87.5 |
| Perc. 95 | 85.7 | 83.3 | 83.3 | 81.3 |  | Perc. 95 | 92.9 | 87.5 | 83.3 | 90.6 |
|  |  |  |  |  |  |  |  |  |  |  |
| Urban, Female, >50 years, Low education (N=20) | | | | |  | Urban, Female, >50 years, Middle education (N=25) | | | | |
|  | Physical | Psychological | Social | Environment |  |  | Physical | Psychological | Social | Environment |
| Mean | 65.6 | 62.5 | 59.7 | 59.4 |  | Mean | 66.0 | 65.9 | 61.2 | 58.8 |
| SD | 12.4 | 12.7 | 12.2 | 10.5 |  | SD | 12.1 | 10.7 | 10.2 | 11.7 |
| Perc. 5 | 48.2 | 41.7 | 41.7 | 46.9 |  | Perc. 5 | 46.4 | 50.0 | 50.0 | 40.6 |
| Perc. 10 | 50.0 | 45.8 | 45.8 | 46.9 |  | Perc. 10 | 50.0 | 50.0 | 50.0 | 46.9 |
| Perc. 25 | 55.4 | 54.2 | 50.0 | 50.0 |  | Perc. 25 | 58.9 | 58.3 | 50.0 | 50.0 |
| Perc. 50 | 67.9 | 62.5 | 62.5 | 59.4 |  | Perc. 50 | 67.9 | 70.8 | 62.5 | 59.4 |
| Perc. 75 | 75.0 | 70.8 | 66.7 | 65.6 |  | Perc. 75 | 75.0 | 70.8 | 66.7 | 67.2 |
| Perc .90 | 82.1 | 75.0 | 75.0 | 73.4 |  | Perc .90 | 82.1 | 75.0 | 75.0 | 75.0 |
| Perc. 95 | 87.5 | 83.3 | 83.3 | 79.7 |  | Perc. 95 | 85.7 | 79.2 | 75.0 | 75.0 |
|  |  |  |  |  |  |  |  |  |  |  |
| Urban, Female, >50 years, High education (N=7) | | | | |  | Urban, Male, 17-30 years, Low education (N=24) | | | | |
|  | Physical | Psychological | Social | Environment |  |  | Physical | Psychological | Social | Environment |
| Mean | 64.8 | 64.9 | 60.7 | 64.3 |  | Mean | 70.5 | 64.9 | 61.6 | 59.9 |
| SD | 10.8 | 17.5 | 16.5 | 15.3 |  | SD | 13.7 | 13.1 | 15.2 | 12.3 |
| Perc. 5 | 46.4 | 41.7 | 50.0 | 50.0 |  | Perc. 5 | 53.6 | 50.0 | 41.7 | 46.9 |
| Perc. 10 | 46.4 | 41.7 | 50.0 | 50.0 |  | Perc. 10 | 53.6 | 50.0 | 50.0 | 46.9 |
| Perc. 25 | 57.1 | 54.2 | 50.0 | 50.0 |  | Perc. 25 | 64.3 | 54.2 | 50.0 | 50.0 |
| Perc. 50 | 64.3 | 58.3 | 50.0 | 59.4 |  | Perc. 50 | 73.2 | 64.6 | 58.3 | 60.9 |
| Perc. 75 | 75.0 | 79.2 | 75.0 | 81.3 |  | Perc. 75 | 78.6 | 72.9 | 75.0 | 67.2 |
| Perc .90 | 78.6 | 91.7 | 91.7 | 87.5 |  | Perc .90 | 89.3 | 83.3 | 83.3 | 75.0 |
| Perc. 95 | 78.6 | 91.7 | 91.7 | 87.5 |  | Perc. 95 | 92.9 | 91.7 | 83.3 | 78.1 |
|  |  |  |  |  |  |  |  |  |  |  |
| Urban, Male, 17-30 years, Middle education (N=66) | | | | |  | Urban, Male, 17-30 years, High education (N=23) | | | | |
|  | Physical | Psychological | Social | Environment |  |  | Physical | Psychological | Social | Environment |
| Mean | 71.7 | 70.1 | 65.8 | 59.2 |  | Mean | 71.4 | 64.7 | 68.1 | 64.1 |
| SD | 12.3 | 13.9 | 16.9 | 14.4 |  | SD | 10.9 | 15.7 | 11.1 | 12.9 |
| Perc. 5 | 50.0 | 45.8 | 41.7 | 34.4 |  | Perc. 5 | 53.6 | 33.3 | 50.0 | 40.6 |
| Perc. 10 | 57.1 | 50.0 | 41.7 | 40.6 |  | Perc. 10 | 57.1 | 41.7 | 58.3 | 40.6 |
| Perc. 25 | 60.7 | 58.3 | 50.0 | 50.0 |  | Perc. 25 | 64.3 | 54.2 | 58.3 | 59.4 |
| Perc. 50 | 71.4 | 75.0 | 66.7 | 56.3 |  | Perc. 50 | 71.4 | 66.7 | 66.7 | 65.6 |
| Perc. 75 | 78.6 | 79.2 | 75.0 | 68.8 |  | Perc. 75 | 78.6 | 75.0 | 75.0 | 75.0 |
| Perc .90 | 85.7 | 87.5 | 91.7 | 78.1 |  | Perc .90 | 85.7 | 79.2 | 83.3 | 78.1 |
| Perc. 95 | 96.4 | 87.5 | 91.7 | 81.3 |  | Perc. 95 | 85.7 | 83.3 | 83.3 | 81.3 |
|  |  |  |  |  |  |  |  |  |  |  |
| Urban, Male, 31-50 years, Low education (N=31) | | | | |  | Urban, Male, 31-50 years, Middle education (N=67) | | | | |
|  | Physical | Psychological | Social | Environment |  |  | Physical | Psychological | Social | Environment |
| Mean | 72.9 | 72.4 | 65.2 | 61.2 |  | Mean | 70.0 | 67.3 | 67.1 | 58.8 |
| SD | 12.3 | 15.7 | 17.9 | 16.7 |  | SD | 10.9 | 12.8 | 16.9 | 13.9 |
| Perc. 5 | 53.6 | 50.0 | 41.7 | 37.5 |  | Perc. 5 | 57.1 | 45.8 | 41.7 | 34.4 |
| Perc. 10 | 53.6 | 58.3 | 50.0 | 46.9 |  | Perc. 10 | 57.1 | 54.2 | 50.0 | 40.6 |
| Perc. 25 | 64.3 | 66.7 | 58.3 | 50.0 |  | Perc. 25 | 64.3 | 58.3 | 58.3 | 50.0 |
| Perc. 50 | 75.0 | 79.2 | 66.7 | 62.5 |  | Perc. 50 | 67.9 | 66.7 | 66.7 | 59.4 |
| Perc. 75 | 82.1 | 79.2 | 75.0 | 71.9 |  | Perc. 75 | 78.6 | 75.0 | 83.3 | 71.9 |
| Perc .90 | 89.3 | 87.5 | 83.3 | 78.1 |  | Perc .90 | 85.7 | 83.3 | 91.7 | 78.1 |
| Perc. 95 | 92.9 | 91.7 | 91.7 | 81.3 |  | Perc. 95 | 92.9 | 87.5 | 91.7 | 81.3 |
|  |  |  |  |  |  |  |  |  |  |  |
| Urban, Male, 31-50 years, High education (N=17) | | | | |  | Urban, Male, >50 years, Low education (N=16) | | | | |
|  | Physical | Psychological | Social | Environment |  |  | Physical | Psychological | Social | Environment |
| Mean | 67.2 | 66.4 | 63.7 | 57.7 |  | Mean | 75.0 | 72.5 | 68.3 | 67.7 |
| SD | 14.0 | 13.1 | 17.4 | 16.4 |  | SD | 11.5 | 13.5 | 14.8 | 16.8 |
| Perc. 5 | 50.0 | 37.5 | 50.0 | 34.4 |  | Perc. 5 | 53.6 | 41.7 | 41.7 | 28.1 |
| Perc. 10 | 53.6 | 50.0 | 50.0 | 37.5 |  | Perc. 10 | 60.7 | 58.3 | 50.0 | 50.0 |
| Perc. 25 | 57.1 | 58.3 | 50.0 | 50.0 |  | Perc. 25 | 69.6 | 62.5 | 54.2 | 56.3 |
| Perc. 50 | 60.7 | 70.8 | 58.3 | 53.1 |  | Perc. 50 | 71.4 | 70.8 | 75.0 | 68.8 |
| Perc. 75 | 75.0 | 79.2 | 75.0 | 62.5 |  | Perc. 75 | 82.1 | 79.2 | 75.0 | 82.8 |
| Perc .90 | 96.4 | 83.3 | 100.0 | 84.4 |  | Perc .90 | 85.7 | 91.7 | 83.3 | 84.4 |
| Perc. 95 | 96.4 | 83.3 | 100.0 | 90.6 |  | Perc. 95 | 100.0 | 95.8 | 100.0 | 90.6 |
|  |  |  |  |  |  |  |  |  |  |  |
| Urban, Male, >50 years, Middle education (N=26) | | | | |  | Urban, Male, >50 years, High education (N=7) | | | | |
|  | Physical | Psychological | Social | Environment |  |  | Physical | Psychological | Social | Environment |
| Mean | 68.2 | 66.3 | 63.3 | 55.8 |  | Mean | 65.8 | 60.7 | 53.6 | 47.8 |
| SD | 9.5 | 8.7 | 12.5 | 9.0 |  | SD | 7.7 | 7.2 | 17.9 | 10.5 |
| Perc. 5 | 50.0 | 54.2 | 41.7 | 40.6 |  | Perc. 5 | 53.6 | 50.0 | 25.0 | 34.4 |
| Perc. 10 | 53.6 | 54.2 | 50.0 | 40.6 |  | Perc. 10 | 53.6 | 50.0 | 25.0 | 34.4 |
| Perc. 25 | 60.7 | 54.2 | 50.0 | 46.9 |  | Perc. 25 | 60.7 | 54.2 | 41.7 | 40.6 |
| Perc. 50 | 67.9 | 64.6 | 62.5 | 53.1 |  | Perc. 50 | 64.3 | 62.5 | 50.0 | 43.8 |
| Perc. 75 | 75.0 | 75.0 | 75.0 | 62.5 |  | Perc. 75 | 75.0 | 66.7 | 75.0 | 56.3 |
| Perc .90 | 78.6 | 75.0 | 75.0 | 68.8 |  | Perc .90 | 75.0 | 70.8 | 75.0 | 65.6 |
| Perc. 95 | 82.1 | 75.0 | 75.0 | 68.8 |  | Perc. 95 | 75.0 | 70.8 | 75.0 | 65.6 |
| Perc: Percentile  *: Low education, means primary school and below, middle education means high school, and high education is college/university | | | | | | | | | | |
